# Identification of CDPKs involved in TaNOX7 mediated ROS production in wheat

**DOI:** 10.3389/fpls.2022.1108622

**Published:** 2023-01-23

**Authors:** Chun-Hong Hu, Bin-Bin Li, Peng Chen, Hai-Yan Shen, Wei-Gang Xi, Yi Zhang, Zong-Hao Yue, Hong-Xing Wang, Ke-Shi Ma, Li-Li Li, Kun-Ming Chen

**Affiliations:** ^1^ College of Life Science and Agronomy, Zhoukou Normal University, Zhoukou, China; ^2^ State Key Laboratory of Crop Stress Biology in Arid Areas, College of Life Sciences, Northwest A&F University, Yangling, Shaanxi, China; ^3^ Key Laboratory of Plant Genetics and Molecular Breeding, Zhoukou Normal University, Zhoukou, China

**Keywords:** wheat (*Triticum aestivum*), TaCDPK protein, identification, expression analysis, TaCDPK2/4-TaNOX7 interactions; ROS production

## Abstract

As the critical sensors and decoders of calcium signal, calcium-dependent protein kinase (CDPK) has become the focus of current research, especially in plants. However, few resources are available on the properties and functions of CDPK gene family in *Triticum aestivum* (TaCDPK). Here, a total of 79 *CDPK* genes were identified in the wheat genome. These *TaCDPKs* could be classified into four subgroups on phylogenesis, while they may be classified into two subgroups based on their tissue and organ-spatiotemporal expression profiles or three subgroups according to their induced expression patterns. The analysis on the signal network relationships and interactions of TaCDPKs and NADPH (reduced nicotinamide adenine dinucleotide phosphate oxidases, NOXs), the key producers for reactive oxygen species (ROS), showed that there are complicated cross-talks between these two family proteins. Further experiments demonstrate that, two members of TaCDPKs, TaCDPK2/4, can interact with TaNOX7, an important member of wheat NOXs, and enhanced the TaNOX7-mediated ROS production. All the results suggest that TaCDPKs are highly expressed in wheat with distinct tissue or organ-specificity and stress-inducible diversity, and play vital roles in plant development and response to biotic and abiotic stresses by directly interacting with TaNOXs for ROS production.

## 1 Introduction

In multicellular organisms, including plants calcium ion (Ca^2+^) is recognized as a vital and conserved secondary messenger that is necessary for signaling transduction. As the Ca^2+^ sensors and responders, calcium-dependent protein kinase CDPKs/CPKs universally present in green algae, oomycetes, protists, especially in higher plants, but absent in animals and fungi (Valmonte et al., 2014), while CDPK-related receptor-like kinases CRKs, that share some conserved homology from the parent CDPKs, are only observed in plants (Hrabak et al., 2003). For example, 34 AtCDPKs and 8 CRKs in *Arabidopsis* (*Arabidopsis Thaliana*) (Yip Delormel and Boudsocq, 2019), 29 OsCDPKs in rice (*Oryza sativa *L.) (Asano et al., 2005), 42 ZmCPKs in maize (*Zea mays*) (Li et al., 2018), 44 BaCDPKs in banana (*Musa paradisiaca*) (Li et al., 2020), 30 PtCDPKs in black cottonwood (*Populus trichocarpa*) (Zuo et al., 2013), 128 WmCDPKs and WmCRKs in watermelon (*Citrullus lanatus*) (Wei et al., 2019), and so on, were all identified and described in plants. CDPKs play important roles in many biological processes, such as growth and development, physiological regulation and control, and response to biotic and abiotic stresses in plants. For example, in *Arabidopsis*, AtCPK1 was found to be involved in the regulation of cell death by phosphorylating the senescence master regulator ORE1, a NAC transcription factor also called AtNAC2/ANAC092 (Durian et al., 2020); AtCPK12 performed a negative ABA-signaling regulator functions in seed germination and post-germination growth (Zhao et al., 2011); AtCPK33 plays an important role in strigolactones (SLs) induced stomatal closure (Wang et al., 2019a). In rice, OsCDPK5/13, as the negative regulators, participate in aerenchyma formation of roots (Yamauchi et al., 2017). While, OsCPK12 performs the positive effect on delaying leaf senescence and providing the potential productivity in plants (Wang et al., 2019b). In okra (*Abelmoschus esculentus* L.), AeCDPK6 can prolong full-blooming period by regulating hyperoside biosynthesis indirectly (Yang et al., 2020a). Moreover, AtCPK28 is not only involved in the regulation of stem elongation and secondary growth (Matschi et al., 2013), but also acts as a negative regulator and plays a crucial role in immune signaling (Monaghan et al., 2014). Similarly, GmCDPK38 also plays a dual role in coordinating flowering time regulation and insect resistance of soybean (*Glycine max*) (Li et al., 2022a). In addition, AtCPK5 directly phosphorylates AtLYK5, a lysin motif receptor-like kinases, and regulates chitin-induced defense responses in *Arabidopsis* (Huang et al., 2020). In terms of abiotic stress, AtCPK12 is involved in plant adaptation to salt stress by regulating Na^+^ and H_2_O_2_ homeostasis (Zhang et al., 2018); StCDPK32 positively modulates physiological properties and photosynthesis in response to salinity stress in potato (*Solanum tuberosum*) (Zhu et al., 2021); overexpression of GmCDPK3 improved soybean tolerance to drought and salt stresses (Wang et al., 2019c). Whereas, PheCDPK22 functions as a negative regulator of drought stress in moso bamboo (*Phyllostachys edulis*) (Wu et al., 2020).

CDPKs also participate in many biological signaling networks in plants, especially by interacting with NOX (also called respiratory burst oxidase homolog, RBOH/Rboh) family proteins, the key producers of reactive oxygen species (ROS) of plants. For example, StCDPK5 directly activates and phosphorylates StRbohB in a calcium-dependent manner to regulate the oxidative burst for defense responses to pathogens (Kobayashi et al., 2007); AtCPK5 phosphorylates AtRbohD and thereby enhances ROS production for defense responses and bacterial resistance (Dubiella et al., 2013). In addition, BnaCPK6L was reported to play an important role in ROS accumulation and hypersensitive response (HR)-like cell death by interacting with and phosphorylating BnaRbohD (Pan et al., 2019). Additionally, StCDPK23 may participate in the wound healing of potato tubers by regulating StRbohs for H_2_O_2_ production (Ma et al., 2022). Intriguingly, OsCPK12 promotes the tolerance of rice to salt stress by repressing the expression level of OsRbohI and reducing the accumulation of ROS (Asano et al., 2012; Boudsocq and Sheen, 2013). More importantly, a MtCDPK5 can directly phosphorylate three Rbohs MtRbohB, MtRbohC, and MtRbohD respectively, which can trigger immune responses to regulating rhizobial colonization in symbiotic cells of barrel medic (*Medicago Truncatula*) (Yu et al., 2018a). Conversely, an OsRbohH can be stimulated by two CDPKs CDPK5 and CDPK13 for ROS production, which is essential for aerenchyma formation in rice roots (Yamauchi et al., 2017). In wheat (*Triticum aestivum*), our previous studies showed that TaCDPK13 directly interacts with and activates TaNOX7 for ROS production for plant fertility regulation and drought tolerance (Hu et al., 2020a). Besides these, NADPH oxidases TaNOXs, as the key producers of ROS, play crucial roles in various biological processes in plants (Hu et al., 2018). All the results mentioned above prompt us to speculate that there may also be complicated interactions between TaCDPKs and TaNOXs family members in wheat.

However, as of today, only a few of CDPKs in wheat were characterized according to their gene evolutionary (Geng et al., 2011) and expressional characteristics (Martínez-Noël et al., 2007), the functions and signal network relationships of wheat CDPK family genes involved in plant growth regulation and environmental stress response are still largely unknown. In the present study, comprehensive analyses based on bioinformatics approaches and experimental methods were performed to identify the wheat CDPK family genes, their functions and signal network relationships during the plant development and stress response. Based on the results, the interactions between TaCDPK2/4/14/16/20/21 and TaNOX7 were further studied and verified that TaCDPK2/4 can interact with TaNOX7 and co-expression of TaCDPK2/4 with TaNOX7 enhanced ROS production in plants. The results obtained here will largely broaden our understanding of the roles of TaCDPKs and the signal network relationships between TaNOXs and TaCDPKs in wheat.

## 2 Materials and methods

### 2.1 Sequence retrieval and identification of the CDPK gene family in wheat

**Figure 1:**
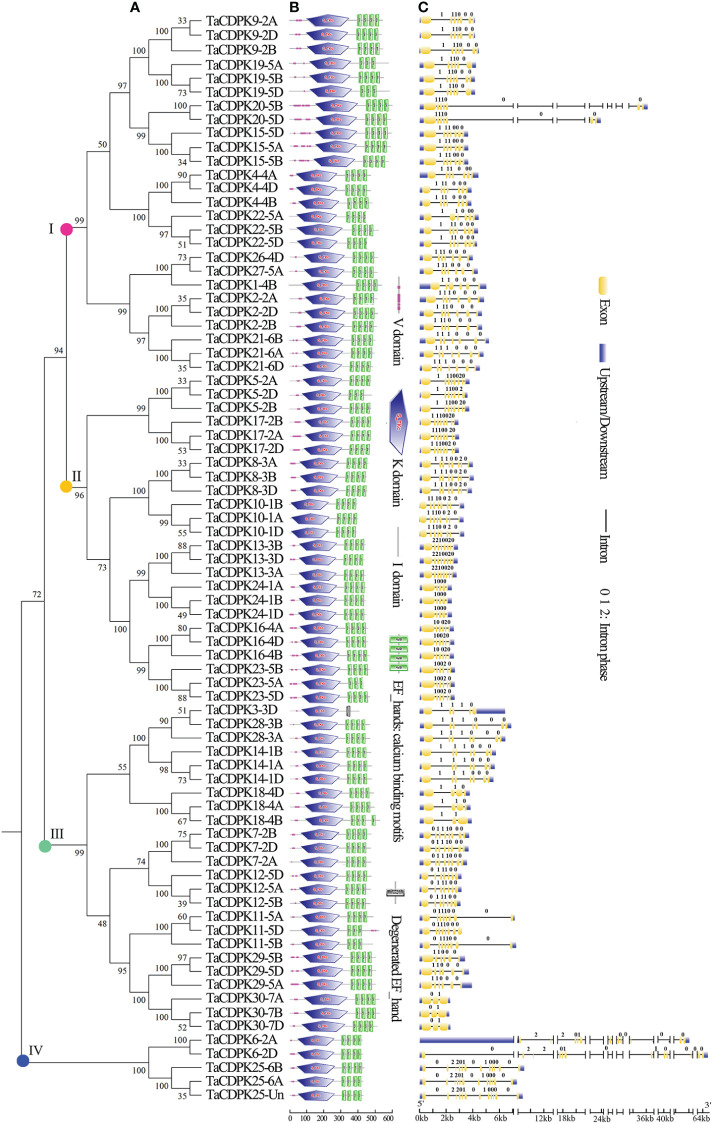
Phylogenetic relationship, domain organization, and exon/intron structure analysis of CDPK family members in wheat. **(A)** The unrooted maximum-likelihood phylogenetic tree of TaCDPK family members were made with MEGA 6.06. Numbers above the nodes represent bootstrap values from 1,000 replications. **(B)** Domain organization of the TaCDPKs. The logos of domain organization were obtained from EMBL-EBI and SMART websites and were amended with Adobe_Photoshop_CS6. The domains: V represents variable domain; K represents catalytic domain; I represents auto-inhibitory domain; C represents the region of calcium binding motifs: EF_hands. **(C)** The exon/intron structures of CDPK family genes in wheat. The numbers 0, 1, and 2 represent the phase of each intron in the sequence.

We retrieved the potential sequences of *CDPK* members in wheat from IWGSC (http://www.wheatgenome.org/, last accessed May 25, 2021), NCBI (https://www.ncbi.nlm.nih.gov/, last accessed May 20, 2021), and Ensembl Plants (http://plants.ensembl.org/Triticum_aestivum/Info/Index, last accessed May 20, 2021) websites, with the well-known *CDPK* sequences as queries. We identified each *CDPK* member by predicting the conserved domains. For further information, we analyzed some physicochemical parameters, predicted the subcellular localization and the numbers of transmembrane helix, and performed amino acid sequence alignment (the detailed information in **Table S1** and **Figure 1**).

### 2.2 Sequence alignment and protein structure analysis

The phylogenetic tree of wheat CDPK family members was constructed with MEGA 6.06 (**Figure 1A**). The logos of domain organization were obtained from EMBL-EBI (http://pfam.xfam.org/search#tabview=tab1) or SMART (http://smart.embl-heidelberg.de/) websites and were amended with Adobe_Photoshop_CS6. The four conserved domains: N-terminal variable domain (V), kinase active region (K), Auto-inhibitory domain (I), and calcium binding motif (C) in each CDPK protein sequence, were generated by MEME suite (http://meme-suite.org/) (**Figure 1B**).

### 2.3 Exon/intron structure analysis and chromosomal location

**Figure 2:**
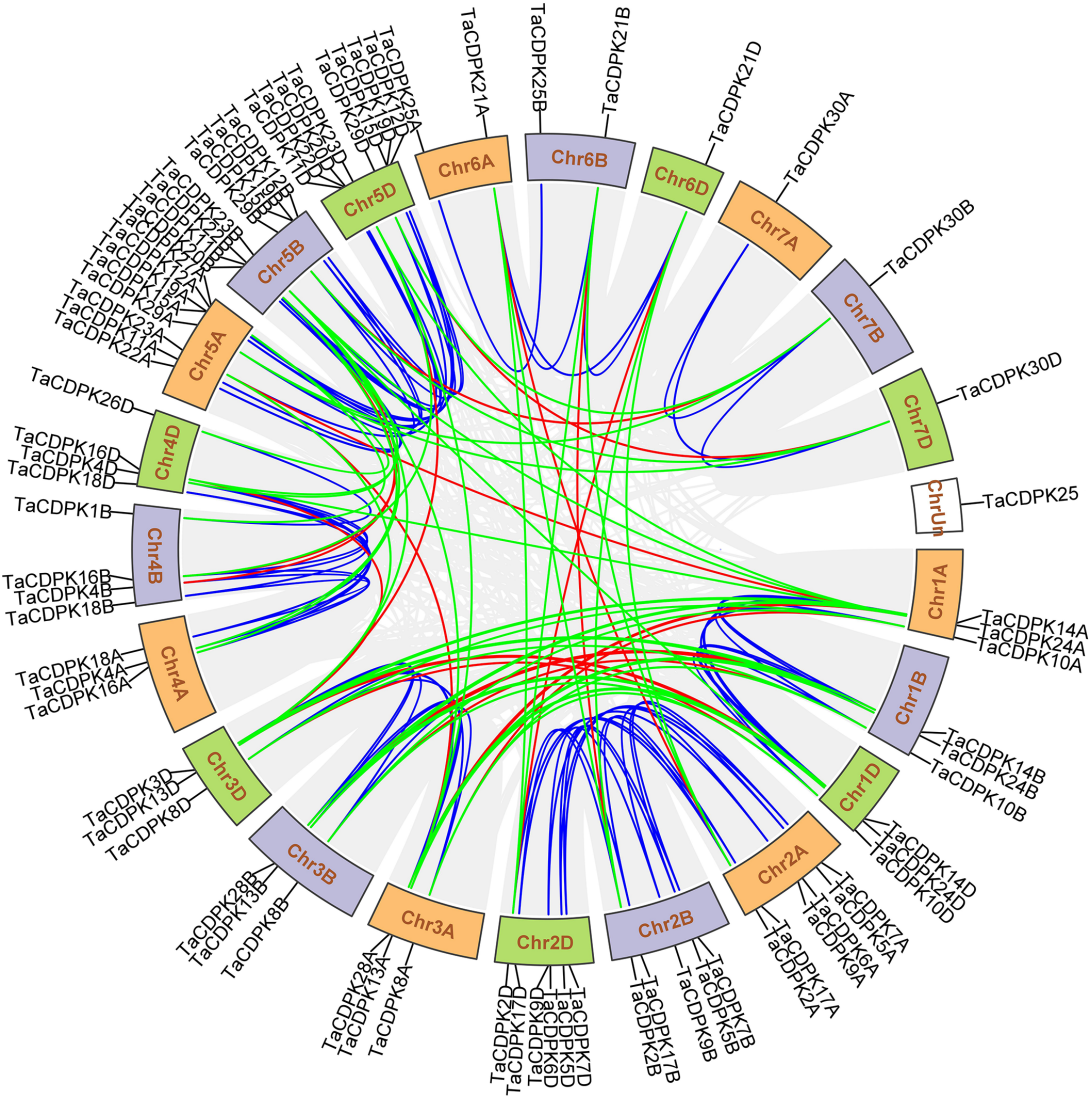
Chromosomal locations of *TaCDPK* genes and their synteny in wheat. Chromosomal locations of *TaCDPK* genes and their synteny are illustrated by the circos diagram. Colored lines indicate similarity. The blue line shows the synteny between homologous genes on homologous chromosomes from different subgenome, such as Chr1A, 1B and 1D; The green lines represent the synteny between homologous genes on non-homologous chromosomes from the same subgenome, such as Chr1A-3A, 1A-5A; The red line shows the synteny of homologous genes between non-homologous chromosomes from different subgenome, such as Chr1A-3B and Chr1A-4D.

The exon/intron logos of individual *CDPK* genes were obtained from the Gene Structure Display Server (http://gsds.cbi.pku.edu.cn) by aligning the coding or cDNA sequences with their corresponding genomic DNA sequences (for the detailed information in **Figure 1C**). The chromosomal distributions of 79 candidate genes *TaCDPKs* were displayed using TBtools software (https://www.yuque.com/cjchen/hirv8i/ra35nv). MCScanX and BLASTP were used to analyze gene duplication events of *TaCDPK* genes in the *Triticum aestivum* genome (**Figure 2**). (See the detailed information about the synteny between homologous genes in **Table S2**).

### 2.4 Prediction and functional analysis of cis-regulatory elements

We selected 2,000-bp genomic DNA sequences upstream of the transcriptional start sites of *TaCDPKs* as the promoter sequences to analyze the cis-acting elements using the databases: PlantCARE (http://bioinformatics.psb.ugent.be/webtools/plantcare/html/) according to the method we previously used (Hu et al., 2018).

### 2.5 Signal network relationships analysis between the members of CDPK and NOX family

**Figure 3:**
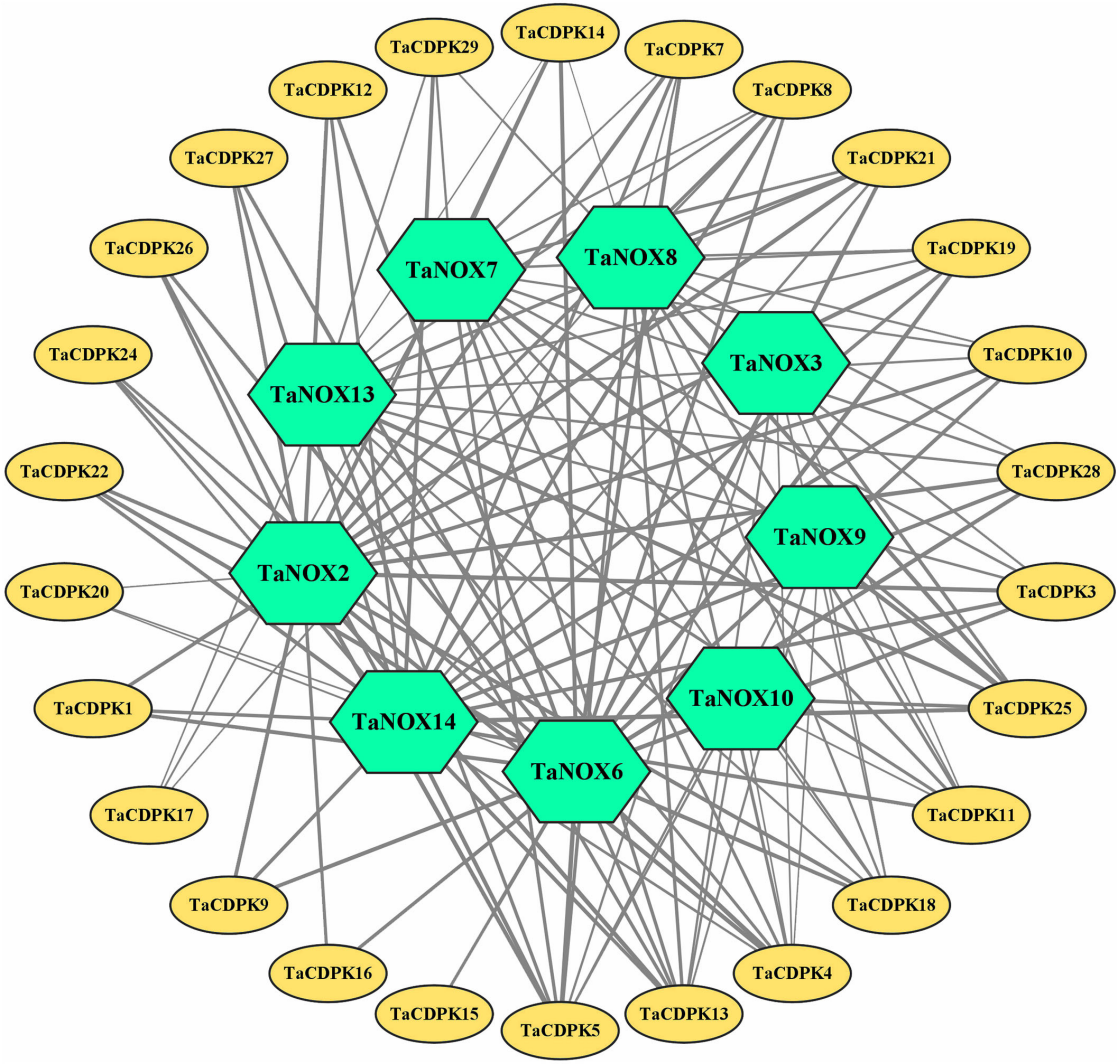
The signal network relationships between the members of CDPK and NOX family. The network signal relationships between the members of CDPK family and NOX family were preliminarily predicted on STRING (http://string-db.org/cgi/input.pl?sessionId=bdYxf9Fv5NiI&input_page_show_search=on), and then the network signal diagram between them were also drawn by softwares of TBtools Cytoscape and Adobe Photoshop. The edge(line) width is positively correlated with the combined score in STRING (Min 1- Max 3).

The signal network relationships between the members of CDPK family, NOX family were drawn by using Cytoscape software and Adobe Photoshop, based on the information from STRING (http://string-db.org/cgi/input.pl?sessionId=bdYxf9Fv5NiI&input_page_show_search=on). (See the detailed information in **Figure 3** and **Table S3**).

### 2.6 Firefly luciferase complementation imaging (LCI) assay

To verify the interaction between TaNOX7 and TaCDPK2/4/14/16/20/21, the firefly luciferase complementation imaging (LCI) assay was performed according to a previously described method (Hu et al., 2020a), with TaCDPK13 as the positive control group. First, we constructed the expression vectors *TaCDPK2*/*4*/*14*/*16*/*20*/*21*-*cLUC* (the C-terminal gene fragment of the Luciferase) and *TaNOX7*-*nLUC* (the N-terminal gene fragment of the Luciferase), then transformed them into wild tobacco (*Nicotiana benthamiana*) leaves by an Agrobacterium-mediated transient transform method mentioned above. After inoculating for 2 or 3 days, the chemiluminescence images and the fluorescence intensity profiles were all taken by a plant living imaging system (Lumazone Pylon2048B, Princeton). The primers used for the vector construction are listed in the Supporting Information (**Table S4**).

### 2.7 Bimolecular fluorescence complementation (BiFC) assay

The bimolecular fluorescence complementation (BiFC) assay was performed with TaCDPK13-TaNOX7 interaction as the positive control group (Hu et al., 2020a), according to the method described by Walter and others (Walter et al., 2004). The coding regions of *TaCDPK2*/*4*/*16* genes were cloned into the pSPYNE vector with the N-terminal gene fragment of the yellow fluorescent protein (*nYFP*), and *TaNOX7* was cloned into the pSPYCE vector with the C-terminal gene fragment of the yellow fluorescent protein (*cYFP*). Then, the *Agrobacterium*-mediated transient transform method was used to transiently coexpress TaNOX7-cYFP and TaCDPK2/4/16-nYFP in *N. benthamiana* leaves. The fluorescence visualization in leaves was ultimately observed with a confocal microscope (A1R, Nikon, Tokyo, Japan). The primers used for the vector construction are listed in the Supporting Information (**Table S4**).

### 2.8 Co-immunoprecipitation (Co-IP) assays

The total proteins were extracted from *N. benthamiana* leaves using a membrane protein extraction method with some modification (Liu et al., 2016). The protein extracts were denatured and separated by SDS-PAGE (sodium dodecyl sulphate-polyacrylamide gel electrophoresis) and then the gel was stained with Coomassie Brilliant Blue. For the co-immunoprecipitation (Co-IP) assay, TaNOX7_(1047 bp)_-GFP-tagged and TaCDPK2/4/13-6*Myc-tagged proteins were detected by monoclonal anti-GFP antibody and anti-Myc antibody (SA003; ABclonal, Wuhan, China), respectively. HRP (horseradish peroxidase) goat anti-mouse IgG antibody (SA003; ABclonal) and antigen-protein complex were detected using the ECL protein gel blot detection kit (GE Healthcare Life Sciences, Beijing, China) and Light-Capture equipped with a CCD camera (ATTO, Shanghai, China) as described by Kobayashi and others (Kobayashi et al., 2007). The primers used for the vector construction are listed in **Supplementary Table S4**. *TaNOX7*_(1047 bp)_ represents the truncated gene sequence from the start codon ATG to the 1047th base of *TaNOX7*, and TaNOX7_(1047 bp)_ includes the conserved functional domain NADPH_Ox and CDPK binding sites (Hu et al., 2018).

### 2.9 Detection of ROS production

The histochemical analyses of H_2_O_2_ accumulation in plant tissues were conducted by 3, 3′- diaminobenzidine (DAB) using an *Agrobacterium*-mediated instantaneous transformation system according to a previously described method (Kumar et al., 2014). The leaves of *N. benthamiana* after agroinfiltrated for 2-3 days were separated from plants and put into DAB staining solutions in darkness at room temperature for several hours. After exposed in light for 2~3 h, the samples were then immersed into bleaching solution (ethanol: acetic acid: glycero = 3:1:1) and boiling in water bath for 10~15 min. The bleaching process was repeated 2~3 times for the clearer photographs.

### 2.10 Subcellular localization analysis

The subcellular location of TaCDPK2/4 and TaNOX7 were examined with *N. benthamiana* as the materials using an *Agrobacterium*-mediated instantaneous transformation system according to the method with some modifications (Chen et al., 2009). The full-length open reading frame of *TaNOX7* and *TaCDPK2*/*4* gene sequences were used to construct fusion expression vectors containing the gene sequences of GFP (green fluorescent protein) or mCherry (red fluorescent protein): pCAMBIA1301-2*35S-*TaNOX7*-*eGFP*, pCAMBIA131-2*35S-*TaCDPK2*/*4*-*mCherry*. At the same time, the membrane protein AtCBL1n-eGFP was used as a positive control, and the constructed expression vector was transformed into tobacco mesophyll cells by transient transformation mediated by *Agrobacterium tumefaciens*. After 60-84 h of co-culture, the leaves were isolated, and the subcellular localization of proteins were observed by using laser confocal (A1R, Nikon, Tokyo, Japan) at 488 nm (eGFP), 561 nm (mCherry) and 637 nm (Chlorophyll) emission wavelengths. The primers used for the vector construction are listed in the Supporting Information (**Table S4**).

### 2.11 Plant materials, treatments, expression profile analysis

Wheat (*T. aestivum* cv. Chinese Spring) seedlings growing in field were harvested from different developmental stages and used for gene cloning and expression profile analysis. For analysis of the inducible expression profiles of the *CDPK* genes, the spikelet at the early stage of wheat flowering infected by *Fusarium graminae* spore with the method of single flower infusion, and the 10 d old hydroponic seedlings treated with 4°C, 200 mM NaCl, 20% polyethylene glycol 6000 (PEG6000), 100 μM methl jasmonic acid (MeJA), 100 μM abscisic acid (ABA), 500 μM salicylic acid (SA), and 50 μM brassinosteroids (BR), respectively, for 0 h, 24 h and/or 48 h, and with 40°C for 0 h, 12 h and/or 24 h, were all used as the materials for RNA extraction using RNAiso TM Plus (Takara, Dalian, China) performance. In addition, tissue-specific expression profiles, inducible expression profiles of *TaCDPK* genes in wheat were performed using bioinformatics methods based on the online database Genevestigator (https://genevestigator.com/gv/) and/or by quantitative real-time PCR (qRT-PCR) with *TaActin* (AB181991.1) and *TaGAPDH* (ABS59297.1) as the internal transcript level controls. All the results mentioned above were presented as heat maps or histograms. All the expression levels represent the mean ± SD of data collected from three independent experiments with each having three or four replicates. The primers used for qRT-PCR are listed in the **Supplementary data** (**Table S4**).

## 3 Results

### 3.1 Identification of CDPK family genes in wheat genome

A Hidden Markov Model (HMM) search was performed to investigate and characterize the *CDPK* gene family in wheat genome, and a total of 79 candidates were identified (**Table S1**). The homologous genes from different subgenomes (A, B, and D) were assigned the same number in gene denomination due to their similarity in gene structure and protein size (**Figure 1**). Intriguingly, the *CDPK* family genes in wheat genome are regularly distributed across the chromosomes (**Figure 2**). It seems that all the predicted *CDPK* candidates are mainly distributed on Chr 5, followed by Chr 2, 4, 1, 3, 6, and 7 in turn. Moreover, the location information of *CDPK25* is unclear, which is referred to as Chr Un. Considering the objective fact that the distributions of homologous genes (such as *TaCDPK2A*/*B*/*C* on Chr 2) are symmetrical on subchromosomes. Therefore, we speculate that *CDPK25* on Chr Un actually distributes on Chr 6D. This prediction was further confirmed by the cluster analysis using protein sequences of CDPK family as reference (**Figure 1**). In addition, the asymmetrical distribution of *CDPK4*/*16*/*18* between Chr 4B/D and A in **Figure 2**, implying that there are orientation errors on chromosome localization. All these anomalies may provide references for the precise localization of *CDPK4*/*16*/*18*-colinked genes.

### 3.2 Gene structure and domain composition

As can be seen in **Figure 1C**, the gene structures are quite diverse between the *TaCDPKs* with different intron numbers and length except for the certain homologous genes from different subgenomes (A, B, and D). Except for the members *TaCDPK6*/*20* (64 Kb and 36 Kb, respectively), the length of different *TaCDPK* genes varies from 2 Kb to 8 Kb. As shown in **Figure 1B**, almost all the members of CDPK family have four conserved domains, namely N-terminal variable domain (V), Catalytic domain (S_TKc)(also known as kinase active region; K), auto-inhibitory domain (I), and calcium binding domain (C) conceived with three or four EF_hand motifs. Based on their sequence homology, all the 79 members of TaCDPKs could be divided into four subgroups I, II, III, and IV. The domain composition of the proteins is also different between the subgroups. For example, the members of TaCDPK22/1/10/13 in subgroup I and II have no variable domains (V); TaCDPK3 in the subgroup III has only one degenerated EF_hand motif, and the variable domain (V) of TaCDPK11-5D is also abnormally present on the C-side. As speculated above, the member TaCDPK25 mapped on Chr Un were clustered together with their homologue TaCDPK25 from Chr 6A/6B, indicating that gene *TaCDPK25*-*Un* may be objectively present on Chr 6D. Surprisingly, the members come from different Chr with different serial numbers are also clustered together, such as TaCDPK26-4D and TaCDPK27-5A. In addition, different numbered members with different structures are also firstly grouped together, such as TaCDPK28-3A/B and TaCDPK3-3D (**Figure 1A**). Taken together, the complexity of genes and protein structures and the confusing clustering relationships imply some complex evolutionary relationships and functional diversity between CDPK family members.

### 3.3 Tissue and spatio-temporal specific expression of *CDPK* family genes in wheat

To clarify the tissue and spatio-temporal expression profiles of *CDPK* family genes during the development of wheat, a set of microarray data for the gene expression was obtained from Genevestigator v3 (**Figures S1, S2**). To simplify the phraseology in the following experiments and optimize the graphs in this paper, the homologous genes located on different subchromosomes (Chr A, B, and C) that are referred to as *TaCDPKx*; for example, *TaCDPK5-2A*, *-2B*, and *-2D* were all named as *TaCDPK5*.

The expression levels of *TaCDPKs* at 10 developmental stages and 43 tissues were presented different expression patterns with some genes dominantly expressed at a certain stage or tissue (**Figures S1, S2**). Comparison between **Figures S1**, **S2** showed that the tissue and spatio-temporal specific expression profiles of 79 genes echo confirm and complement each other. For example, almost all the members in **Figures S1**, **S2** were all divided into two identical groups I and II except for *TaCDPK17*. The members in group I are all widely expressed in whole developmental stages and most of tissues with the highest level of *TaCDPK26*/*27* in endosperm at dough development stage, *TaCDPK16* in ovary at inflorescence emergence stage (Heading stage) and anthesis stage. Furthermore, *TaCDPK9* in group I expressed with the peaking level in awn, anther, and glum at florescence emergence stages. *TaCDPK4*/*10*/*12* are, in turn, expressed at the highest level with *TaCDPK4* in pistio, spikelet, ovary, *TaCDPK10* in pericarp, *TaCDPK12* in coleoptile, floret; while they all expressed with the highest level at anthesis stage. The members in group II were expressed with low level or expressed restrictively in a certain tissue or at a certain stage. *TaCDPK24* is expressed exclusively in anther at inflorescence emergence stage (Heading stage) as well as *TaCDPK30* in embryo at inflorescence emergence stage. Besides these, there are also some contradictions between **Figures S1**, **S2**. For example, in **Figure S1**, the expression peak of *TaCDPK5* is at germinating seeds, *TaCDPK11* is at anthesis stage, *TaCDPK18* is at seeding stage, *TaCDPK17* is at tillering stage, and *TaCDPK3*/*14*/*28* are at stem elongation stage; while, in **Figure S2**, the expression peak of them, in turn, is at seedling, radicle, shoot apex/ovary. Unsurprisingly, the members with high homology at protein sequence, such as *TaCDPK26* and *TaCDPK27*, also have the similar expression patterns, as well as *TaCDPK3* and *TaCDPK28* in **Figures S1**, **S2**. Obviously, the different results in **Figures S1**, **S2** may be attributed to the different experimental materials, which indicating that the results in **Figures S1**, **S2** are complementary as well as confirming each other.

**Figure 4:**
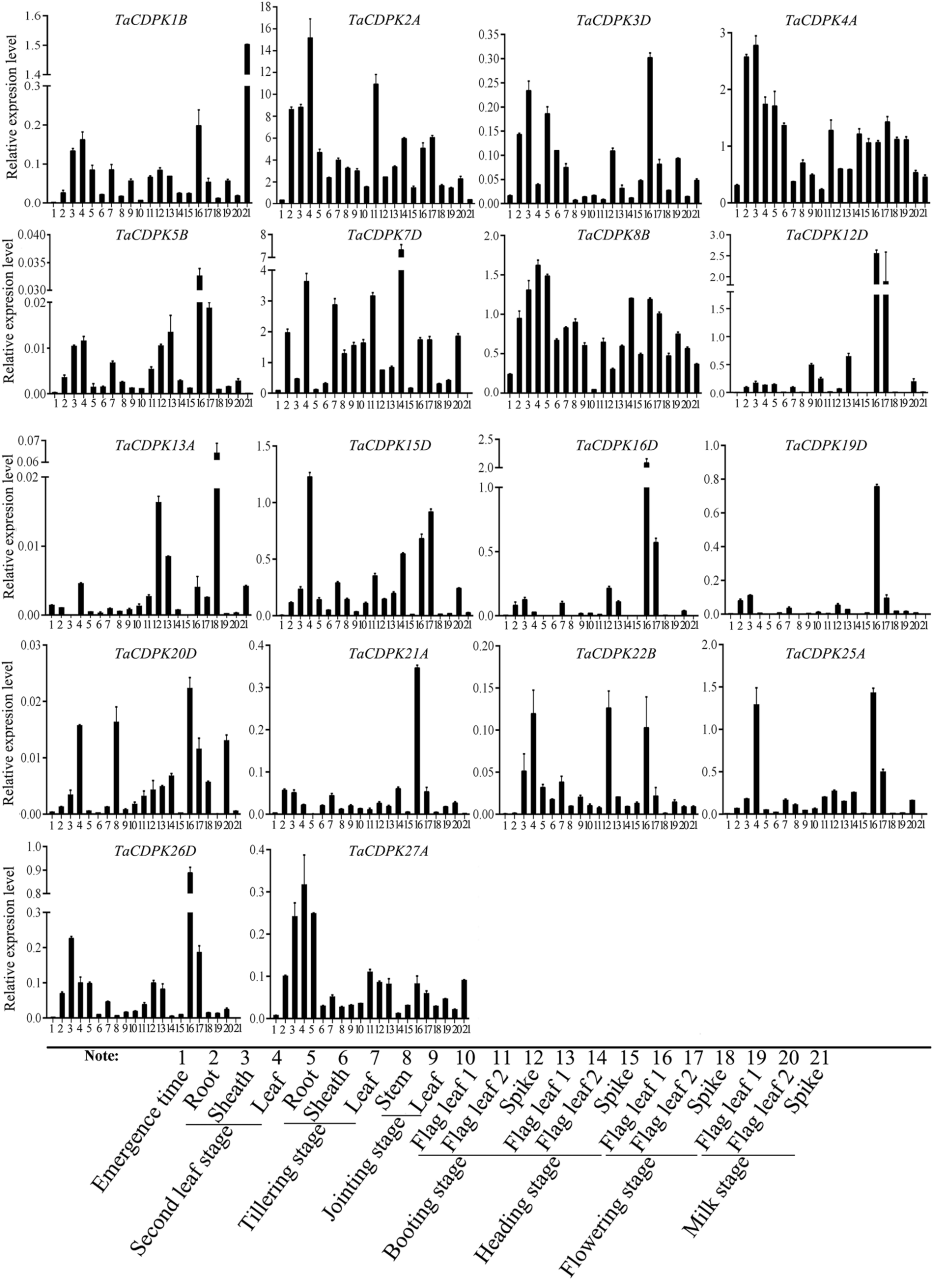
The tissue-specific and spatio-temporal expression profiles of *TaCDPKs* in wheat by qRT-PCR. Expression analysis of *TaCDPKs* in 21 tissues from 8 different development stages of wheat by qRT-PCR. All the expression levels represent the mean ± SD of data collected from the experiments with each having three or four replicates.

In order to further study the expression specificity of *TaCDPKs*, the tissue and spatio-temporal expression profiles were also performed in 21 tissues from 8 different development stages by qRT-PCR (**Figure 4**). Due to the low expression level of *TaCDPK6*/*11*/*18*/*23*/*24* as shown in **Figure S1/S2**, or the nonspecific amplification of *TaCDPK9*/*10*/*14*/*17*/*28*/*29*/*30*, the expression profiles of them could not be obtained here. From **Figure 4**, we can see that every member of *TaCDPKs* has its special expression pattern. For example, *TaCDPK2*, *4*, *7* are expressed with peak in leaf at seedling stage, in sheath at seedling stage, and in flag leaf 1 at heading stage, respectively, though they are all highly expressed in the whole plant. Significantly, the expressive peaks of 9 *TaCDPK* members *TaCDPK3*/*5*/*12*/*16*/*19*/*20*/*21*/*25*/*26*, are all in the flag leaves at flowering stage. *TaCDPK13* is mainly expressed in spikes at milk and heading stages; *TaCDPK8* is expressed in all the tissues with no specialty. In addition, compared with **Figures S1**, **S2**, the expression patterns of *TaCDPKs* in **Figure 4**, are not always consistent. These different results are undoubtedly due to the different experimental methods, sampling period, or growth environment conditions. Therefore, based on previous studies and comprehensive analysis of **Figure S1/S2** and **Figure 4**, we systematically illuminate the unique tissue and developmental expression profiles of *TaCDPK* family members (Shown in **Table 1**).

**Table 1:** Expression specificity and functional diversity of *TaCDPKs* in wheat.

TaCDPKs	Tissue specificty and Expression level	Spatio-temporal pecificty and Expression level	Biotic stress and Expression level	Abiotic stress and Expression level	Response to hormone/ Expression level
*TaCDPK1*	█ Endosperm	█ Milke	█ *F. gra.*		MeJA 5.1
*TaCDPK2*	█ Pistil/Lemma/Yong leaf/Flag Leaf	█ Booting	█ *F. gra.*	█ Cold	BR/MeJA 5.1
*TaCDPK3*	█ Leaf	█ Sheedling, Booting	█ *F. gra.*		MeJA 22 BR 15
*TaCDPK4*	█ Ovary	█ Anthesis, Sheedling	█ Flg22 █ *P. gra*.		MeJA 8.2
*TaCDPK5*	█ Flag leaf	█ Anthesis	█ *F. gra.* █ *P. gra.*	█ Cold	MeJA 1.2
*TaCDPK6*	█ Shoot	█ Germination	█ *F. gra.*	█ Heat	
*TaCDPK7*	█ Lemma, Flag leaf	█ Anthesis	█ *F. gra.*	█ Heat	BR 1000 MeJA 830
*TaCDPK8*	█ Radicle, █ Ovary, Anther	█ Inforescence, Emergence	█ *F. gra.*	█ Heat	BR 1.3
*TaCDPK9*	█ Anther, █ Awn	█ Inforescence, Emergence	█ *F. gra.*	█ Heat	
*TaCDPK10*	█ Lemma, █ pericarp	█ Anthesis	█ Flg22 █ *P. gra*. █ *P. str.*	█ Heat	
*TaCDPK11*	█ Lemma	█ Anthesis		█ Heat	
*TaCDPK12*	█ Flovet, Flag leaf	█ Anthesis	█ *F. gra*. █ Flg22 █ *P. gra.*	█ Heat	BR 350 SA 55 MeJA 50
*TaCDPK13*	█ Anther	█ Anthesis	█ *F. gra.*		ABA 1.0
*TaCDPK14*	█ Shoot apex	█ Stem elongation	█ *P. gra.*		
*TaCDPK15*	█ Floret, Flag leaf, █ Coleoptile	█ Anthesis █ Germination	█ *F. gra.* █ *P. gra.* █ *P. str.*	█ Drought █ NaHS	BR 5.1
*TaCDPK16*	█ Ovary, Flag leaf	█ Anthesis			BR 90 MeJA 60
*TaCDPK17*	█ Radicle	█ Seedling	█ *F. gra.*		
*TaCDPK18*	█ Radicle tip	█ Germination	█ *F. gra.*		
*TaCDPK19*	█ Microspore, Flag leaf	█ Anthesis			MeJA 28 BR 20
*TaCDPK20*	█ Anther	█ Anthesis			BR 0.4
*TaCDPK21*	█ Radicle	█ Tillering	█ *F. gra.*		BR 15
*TaCDPK22*	█ Coleoptile, Spike	█ Inforescence	█ *F.gra.*		MeJA 1.73
*TaCDPK23*	█ Spike, Anther	█ Anthesis			
*TaCDPK24*	█ Anther	█ Inforescence			
*TaCDPK25*	█ Lemma, Flag leaf	█ Anthesis/flowering stage	█ *F. gra.* █ *P. gra.* █ *P. str.*	█ NaHS	BR 32.8
*TaCDPK26*	█ Encosperm	█ Dough	█ *F. gra.*		BR 20
*TaCDPK27*	█ Encosperm	█ Dough	█ *F. gra.*	█ Heat	SA 3.5
*TaCDPK28*	█ Shoot apex █ Ovary	█ Stem elongation	█ *F. gra.*		
*TaCDPK29*	█ Lemma	█ Anthesis			
*TaCDPK30*	█ Embryo	█ Inforescence		█ Dark/Light	
	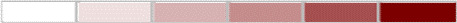 0% Expression level 100%		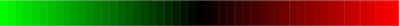 Down-regulated Up-regulated		
**Legends**					


*F. gra.*, *Fusarium graminearum*; Flg22, Flagelin 22; *P. gra.*, *Puccinia graminis*; *P. str.*, *Puccinia striiformis*; NaHS, sodium hydrosulfide; MeJA, methl jasmonic acid; BR, brassinosteroid; SA, salicylic acid; ABA, abscisic acid.

### 3.4 Inducible expression profiles of *TaCDPK* family genes

**Figure 5:**
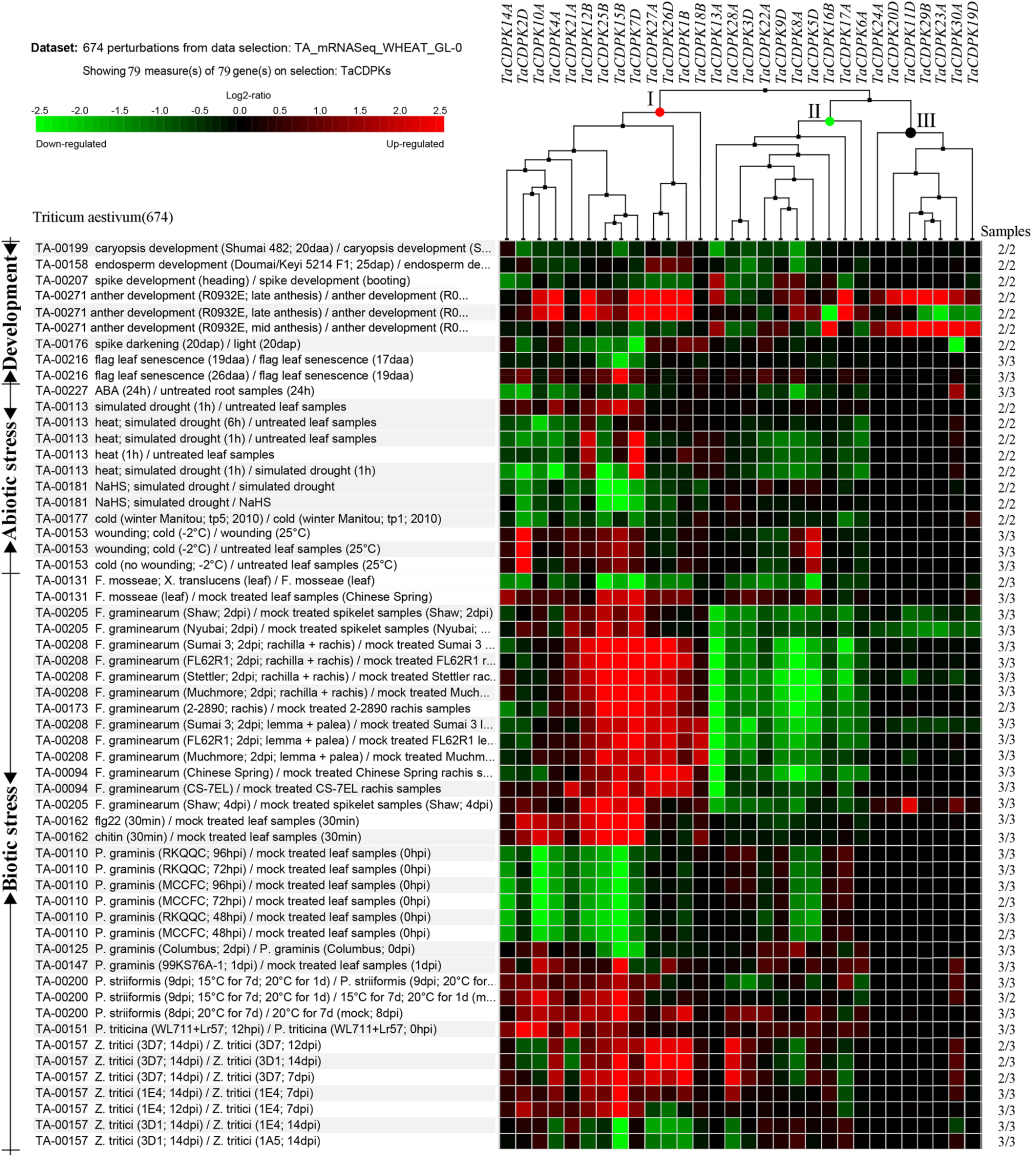
Inducible expression profiles of *TaCDPKs* in wheat. Inducible expression profiles of *TaCDPKs* under a wide of environmental stresses and hormone treatments were selected from the Ta_mRNASeq_WHEAT_GL-0 database in Genevestigator v3. For clear and beautiful presentation of the profiles, each of the 30 members shown in the **Figure 5** contains its homologues (which had been deleted), so there are 79 members in total.

**Figure 6:**
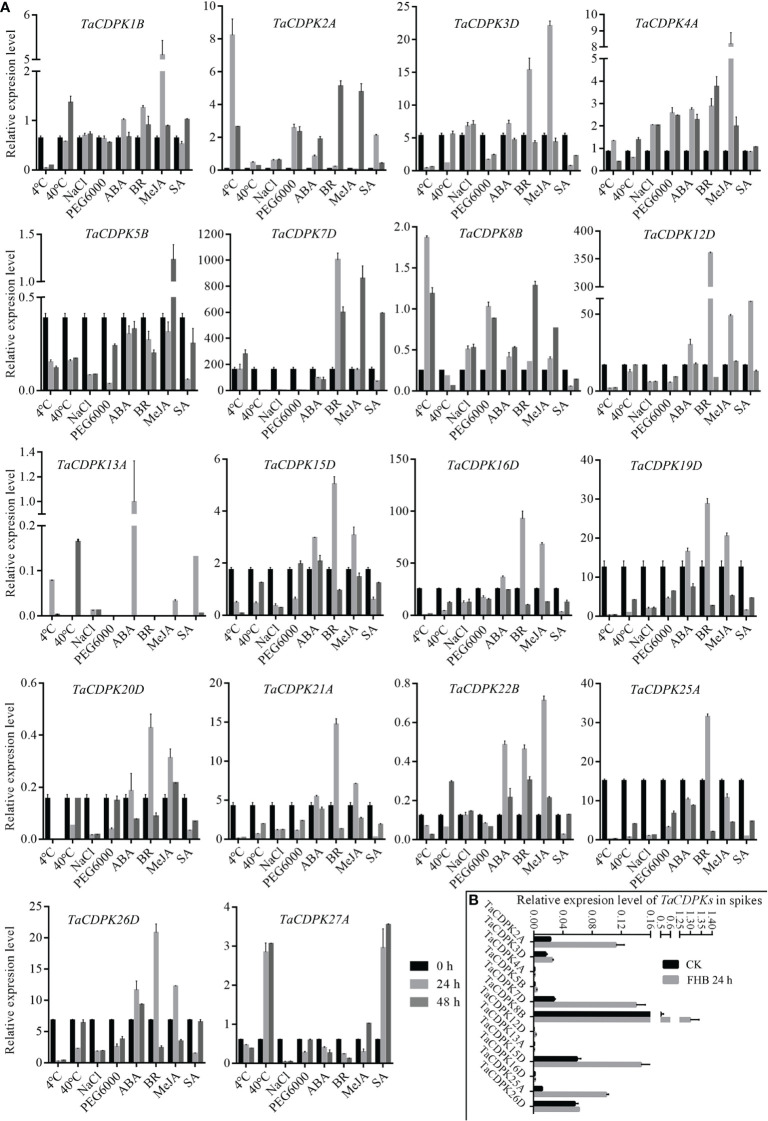
Inducible expression profiles of *TaCDPKs* in wheat by qRT-PCR. **(A)** The inducible expression patterns performed by qRT-PCR under cold (4°C), heat (40°C), 20 % PEG6000, salt (200 mM NaCl), ABA (100 μM), SA (500 μM), MeJA (100 μM) hormone treatments. In all treatments, the heat (40°C) treatment lasted for 12 h and 24 h, respectively, instead of 24 h and 48 h as indicated in the **(A)**. The 10-day old hydroponic seedlings were used for the analysis. **(B)** Inducible expression profiles of *TaCDPKs* in wheat spike treated with *Fusarium graminae* spore suspension for 24 h. The expression level of every gene is the mean of results from three independent experiments, each having three or four replicates. CK: the control group; FHB: the experimental group treated with *Fusarium graminae* spore suspension, which are associated with Fusarium Head Blight (FHB).

To further study the expression characteristics of wheat *CDPK* family genes under suboptimal conditions, we carried out a comprehensive analysis using both the wheat microarray data in Genevestigator v3 (**Figure 5**) and qRT-PCR experiment (**Figure 6**). As can be seen in **Figure 5**, different inducible expression patterns of *CDPK* genes could be seen in responding to different biotic and/or abiotic stresses. According to the expression patterns in **Figure 5**, all the members can be simply classified into three groups: Group I including *TaCDPK1*/*2*/*4*/*7*/*12*/*14*/*15*/*18*/*21*/*25*/*26*, and most of them were upregulated under the biotic stresses except down-regulated under *P. graminis* (*Puccinia graminis*) stress; Group II including *TaCDPK3*/*5*/*6*/*8*/*9*/*13*/*16*/*17*/*22*/*28* were down-regulated under *F. graminearum* (*Fusarium graminearum*); Group III including *TaCDPK11*/*19*/*20*/*23*/*24*/*29*/*30* had no obvious changes under all the biotic or abiotic stresses, but had high expression in another development. Interestingly, these members of Group III also had lower tissue and spatiotemporal expression level comparing with other members in **Figures S1**, **S2**. Furthermore, the high expression level of *TaCDPK2*/*7*/*12*/*15*/*25*/*26* in group I, was further verified under Fusarium Head Blight (FHB) stress in **Figure 6B**. In addition, the expression of most *TaCDPK* genes were significantly up-regulated under the treatment of hormone MeJA and BR (**Figure 6A**), which are consistent with the results that hormone responsive element JARE and ABRE are distributed on almost all the promoters of *TaCDPK* genes (**Figure S3**). Only a few members, such as *TaCDPK27* had obvious response to SA and heat (**Figure 6A**). In order to more intuitively dissect the possible functions of *TaCDPKs*, we listed the specific expressions of each member in plant development or under stress treatment in **Table 1**.

### 3.5 Interaction and co-localization relationships between the members of TaCDPKs and TaNOX7

Lots of studies have addressed that the roles of CDPKs in plant growth regulation and various stress responses are closely associated with NOX-/RBOH mediated ROS production in a Ca^2+^-dependent manner (Potocký et al., 2007; Potocký et al., 2012; Boisson-Dernier et al., 2013; Yamauchi et al., 2017). In addition, numerous literatures confirmed that CDPKs can directly interact with NOXs/RBOHs, and both of which synergistically involved in plant development and response to environmental stress (Kobayashi et al., 2007; Dubiella et al., 2013; Majumdar and Kar, 2018). Therefore, in order to obtain more insights into the function of TaCDPKs, the network signal relationships between 26 members of CDPK family and 9 members of NOX family from the network of STRING had been obtained and drawn with the software of Cytoscape and Adobe Photoshop (**Figure 3**).

**Figure 7:**
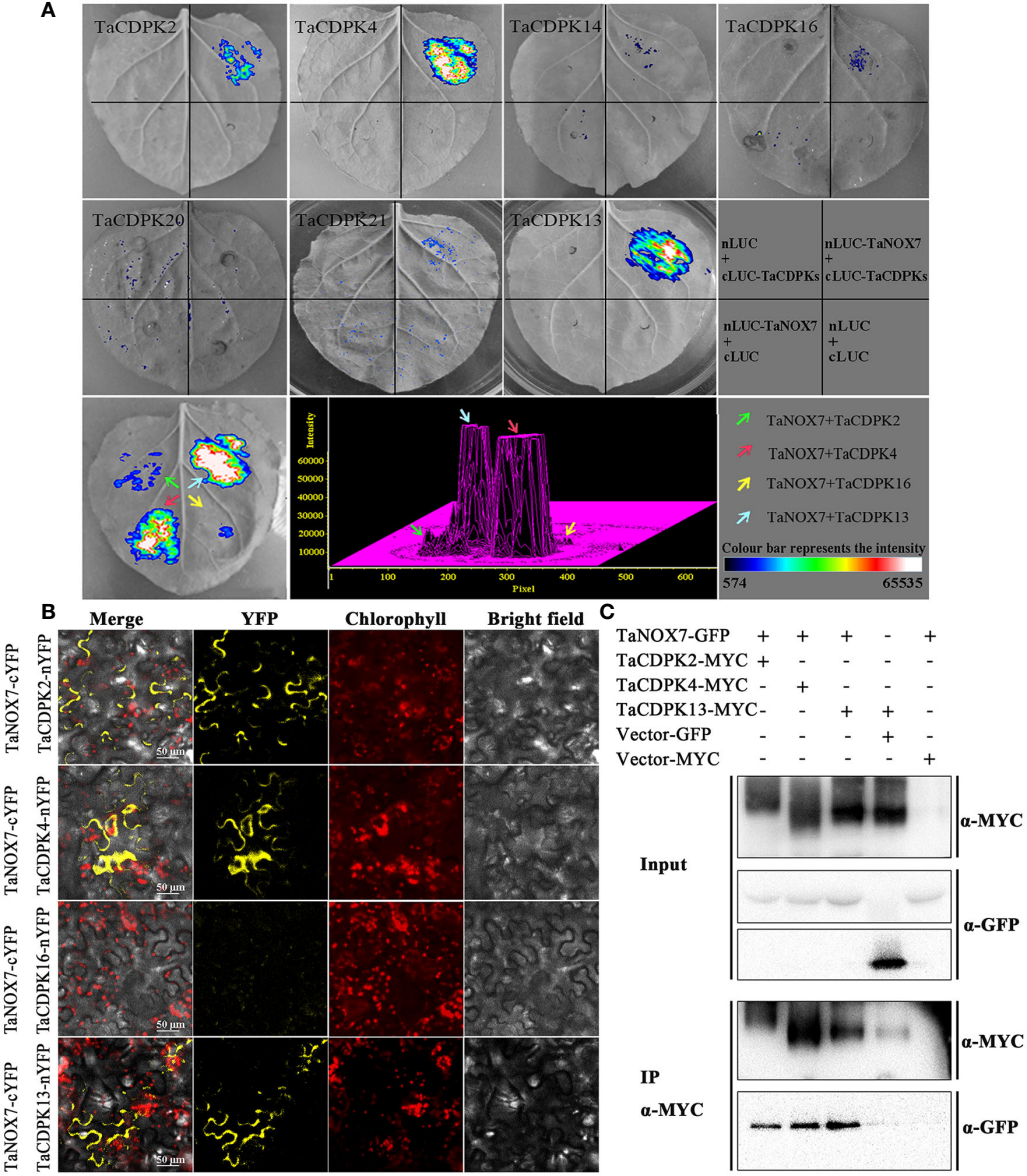
Protein interactions between TaCDPKs and TaNOX7. **(A)** Verification of protein interactions between TaCDPK2/4/14/16/20/21 and TaNOX7 were performed using the method of firefly luciferase complementation imaging (LCI) assay; **(B)** Bimolecular fluorescence complementation **(BiFC)** assay showing the interactions between TaCDPK2/4/16 and TaNOX7. **(C)** The interactions between TaCDPK2/4 and TaNOX7 were confirmed using the assay of co-immunoprecipitation (Co-IP), in which input and immunoprecipitates were analyzed by immunoblotting using anti-GFP and anti-MYC antibodies.

As expected, there are indeed complicated signal relationships between CDPK and NOX family members (**Figure 3**). In addition, our previous research showed that TaCDPK13 could directly interact with and activate TaNOX7 for ROS production, which perform a crucial role during plant development and stress tolerance (Hu et al., 2020a). Therefore, based on the results mentioned above, coupled with the information associated with subcellular localization of TaCDPKs (**Table S1**), we selected TaCDPK2/4/14/16/20/21 as the represents from plasma membrane, cytoplasm, whole cell, chloroplast, and mitochondrion localized members as shown in **Table S1**, and analyzed the relationships between them and TaNOX7 with the physical interaction between TaNOX7 and TaCDPK13 as a positive control. LCI assay showed that there are different intensity of fluorescence signals, presenting TaNOX7 has different interaction with TaCDPK2/4/14/16. The signals from TaCDPK4-TaNOX7 were the strongest, followed by TaCDPK2-TaNOX7, TaCDPK16-TaNOX7, and there are no obvious signals between TaCDPK14/20/21 and TaNOX7 (**Figure 7A**). These indicated that TaNOX7 may interact with TaCDPK2/4/16, respectively, but not with TaCDRK14. Furthermore, as shown in **Figure 7B**, BiFC experiment further verified that TaNOX7 can interact with TaCDPK2/4, but not with TaCDPK16. In addition, Co-IP assays confirmed the conclusion once again (**Figure 7C**). Moreover, the results of subcellular localization indicated that TaCDPK2/4 were all co-located on the cell membrane with TaNOX7 (**Figure S4**), which further provide theoretical support for the TaCDPK2/4-TaNOX7 interaction.

### 3.6 Coexpression of TaNOX7 and TaCDPK2/4 promoted ROS production

**Figure 8:**
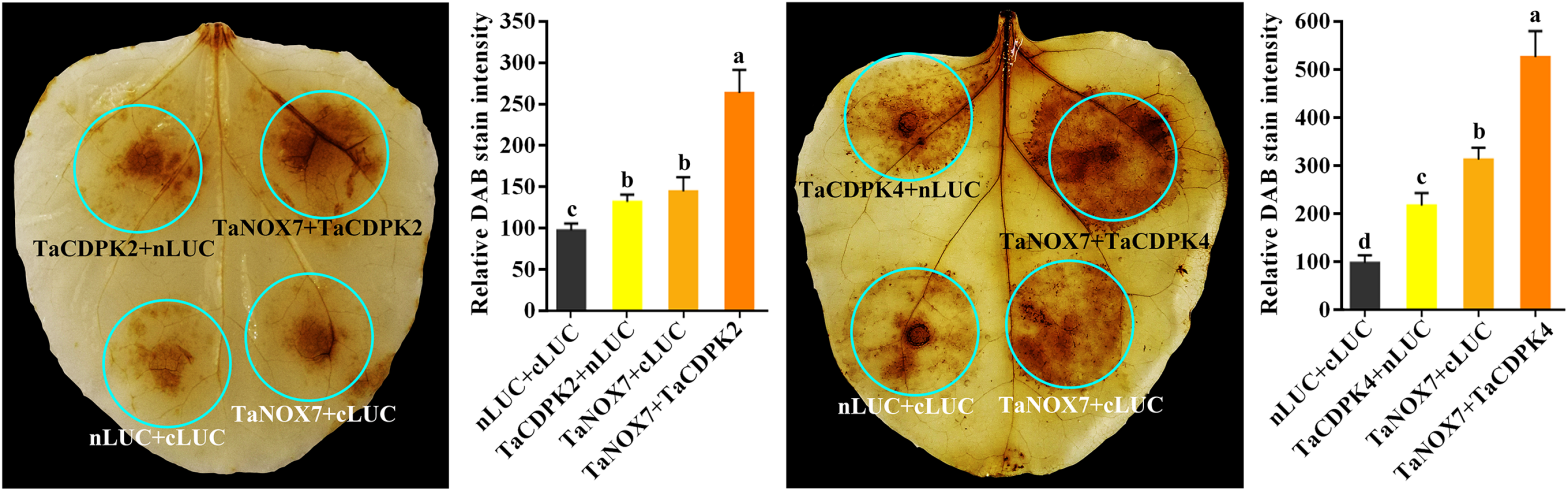
TaCDPK2/4-TaNOX7 interactions enhanced ROS production in plants. Transient coexpression of TaNOX7 with TaCDPK2 or TaCDPK4 all enhanced ROS production in the leaves of *N. benthamiana*. The level of ROS accumulation was detected by the method of DAB (3, 3´- diaminobenzidine) staining method. The DAB staining intensity in *in situ* ROS levels of the agroinfiltrated tobacco leaves was calculated based on the stain intensity of the control “cLUC + nLUC”. Data are means ± SD (n = 10~15 leaves) from more than three independent experiments. Bars annotated with different letters represent values that are significantly different at *P* ≤ 0.05 according to one-way analysis of variance (ANOVA) analysis.

**Figure 9:**
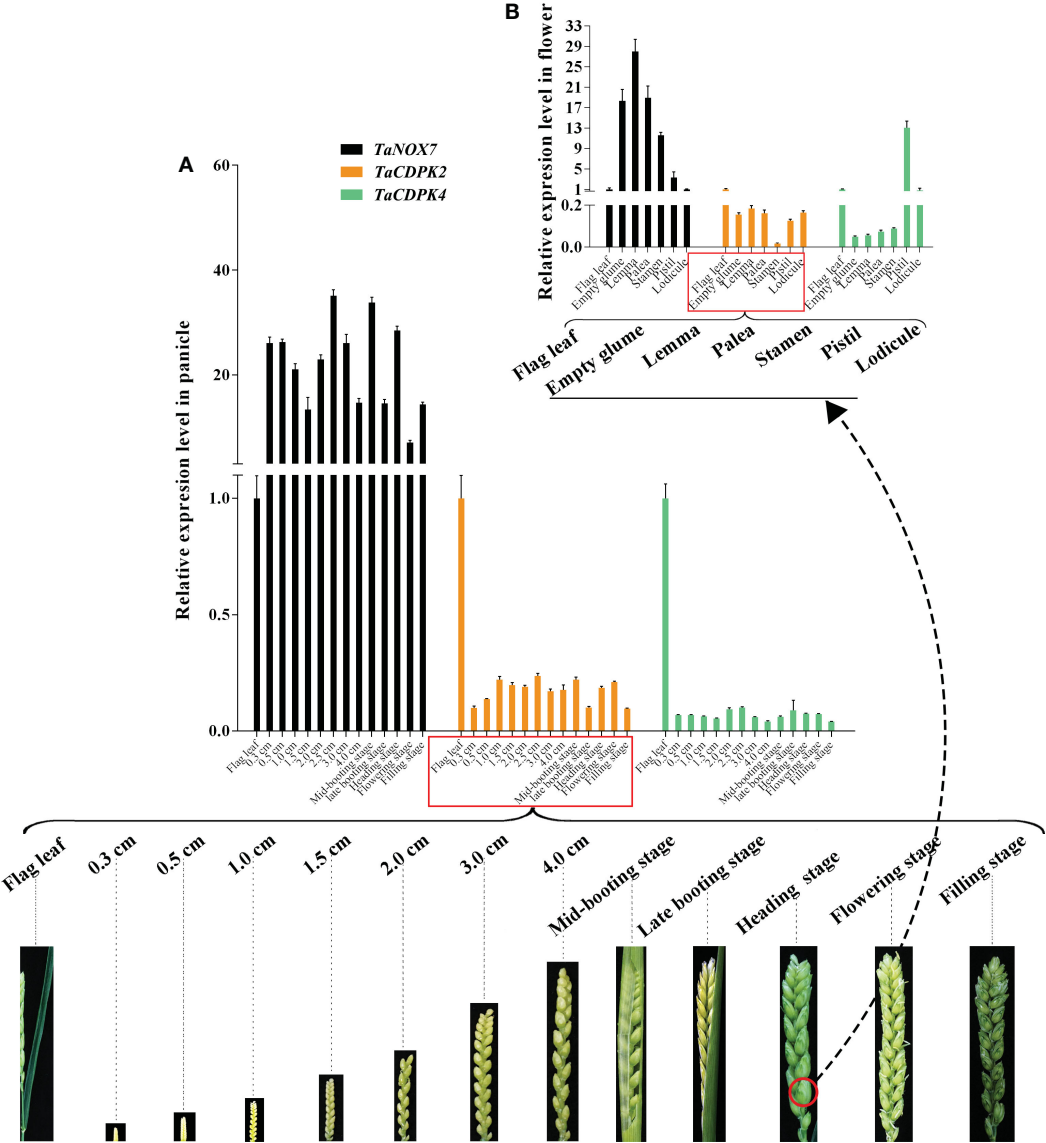
Co-expression interactions between TaNOX7 and TaCDPK2/4 in wheat. **(A)** Co-expression of *TaCDPK2*/*4* and *TaNOX7* in panicles from 12 different developmental stages; **(B)** Co-expression of *TaCDPK2*/*4* and *TaNOX7* in six flower organs at heading stage.

Increasing literatures have reported that CDPK-mediated NOX activation promotes the production of ROS, which plays important role in plants. Consistent with these mentioned above, the results in **Figure 8** showed the red-brown precipitates in the regions that co-expressing of cLUC-TaCDPK2/4 and nLUC-TaNOX7 were significantly higher than those in the control group, which implying that coexpression of TaNOX7 and TaCDPK2/4 promotes ROS accumulation in plant leaves. Hence, what is the biological significance of the interaction between TaCDPK2/4 and TaNOX7? Therefore, we further constructed the sophisticated tissue expression profiles of *TaNOX7* and *TaCDPK2*/*4* at wheat spikes from 12 different developmental stages (**Figure 9A**). As shown in **Figure 9A**, compared with flag leaves, *TaNOX7* was expressed at an absolutely higher level in the young panicles at all the examined stages. Unexpectedly, the expression level of *TaCDPK2*/*4* was much lower in each stage of young panicles than that of flag leaves, which suggesting that there are no co-expression relationships between *TaNOX7* and *TaCDPK2*/*4* during wheat panicle development. Therefore, we further constructed tissue expression profiles of *TaNOX7* and *TaCDPK2*/*4* in six flower organs at heading stage (**Figure 9B**). As shown in **Figure 9B**, the expression level of *TaCDPK2* was still lower than that of flag leaves. Intriguingly, *TaCDPK4* is expressed in the pistils with peak level. These results, coupled with the previous tissue expressions and protein interactions, we concluded that TaCDPK2 was mainly expressed in young leaves and flag leaves, and the interaction with NOX7 might be involved in the vegetative and reproductive growth of plants; TaCDPK4 was mainly expressed in the pistils, and its interaction with NOX7 might contribute to seed development.

## 4 Discussion

### 4.1 Wheat CDPKs are diverse in members and structures with a complicated evolution history

In this study, a total of 79 CDPK family genes, which encode 30 TaCDPKs, were identified according to the sequence analysis and domain composition (**Table S1**). Interestingly, not every protein has three homologous genes distributed on the subchromosomes A, B, and D. For example, *TaCDPK6* includes two homologous genes (*TaCDPK6-6A* and *TaCDPK6-6D*) but *TaCDPK1* has only one (*TaCDPK1-4B*) (**Figure 1A**). These means that their homologs on a certain chromosome might be lost during the long-term evolution and natural selection. In addition, the homologous genes from different subgenomes, such as *TaCDPK27-4D* and *TaCDPK28-5A* in **Figure 1A**, clustered together firstly, which may be attributed to the gene duplication, and/or exon shuffling. Moreover, the phenomenon of motif EF_hand loss is also common in TaCDPK protein sequences. In fact, these anomalies probably belong to genovariation including gene structural variation, rearrangement, DNA sequence loss, and transposon activation occurred frequently during the polyploidization of genome in plants (Jackson and Chen, 2010). Furthermore, degeneration and/or lose of the motif EF_hand in TaCDPKs (such as TaCDPK3) supports the viewpoint that the evolutionary process from TaCDPKs to CDPK-related receptor-like kinases TaCRKs (TaCRKs possess the conserved domains that the typical TaCDPKs have, but lack the EF_hand motifs). In summary, all the information mentioned above, along with the non-random distribution of *TaCDPKs* on 21 chromosomes (**Figure 2**), suggesting that wheat *CDPKs* underwent a complicated evolutionary history, which might endow TaCDPK family with the gene expansion, gene variation, and functional divergence, though further researches are needed to confirm these. In addition, the analyses of gene structure (**Figure 1C**), protein cluster (**Figure 1A**), and gene karyotype (**Figure 2**) indicated that the map of *TaCDPK25* may be mistake, but the corresponding location of *TaCDPK25* on 6D may be more reasonable, which will provide references for the precise mapping of them and their linked genes.

### 4.2 Wheat CDPKs exhibit a great specificity in expression and play vital roles in both the plant growth regulation and stress response

Specific expression is a common characteristic of the genes of a certain protein family in plants, which often reflects the cross-talk and/or difference in the functions of the family members (Hu et al., 2018). In this study, we found that the expression patterns of CDPK family members in wheat showed overall regularity and individual specificity, which indicating their functional synergy and specificity.

Firstly, in terms of organization and spatio-temporal expression, 67.7% of the family members (*TaCDPK2-5*/*7*-*13*/*15*/*16*/*19*/*20*/*22*-*27*/*29*/*30*) all expressed with peak level in different reproductive organs, suggesting that they probably all involved in the regulation of plant reproductive growth but in different reproductive organs (**Figures S1-2**, **4** and **Table 1**). For instance, the expression profile that *TaCDPK13* expressed in anther at the anthesis stage with peak level, which was consistent with our previous results that TaCDPK13 functioned in plant fertility (Hu et al., 2020a). Moreover, increasing reports found that ZmCPK32, AtCPK32, and GmCDPK38 as the homologue of TaCDPK23, played important roles in modulating flowering time and pollen tube growth (Zhou et al., 2014; Li et al., 2018; Li et al., 2022a; Li et al., 2022b), implying that TaCDPK23 probably plays a crucial role in regulating the development of spike/anther at the anthesis stage. OsCDPK1 was proved that it played a functional role in rice seed development (Jiang et al., 2018). As shown in **Table S5**, TaCDPK8 and OsCDPK1 are also the homologues with the highest identification 94.2%, which tempt us to speculate that TaCDPK8 perhaps performs the similar functions to OsCDPK1 by regulating the development of ovary/anther at the inforescence stage in wheat.

Secondly, under abiotic stresses, the expression of *TaCDPK6*-*12* were all sensitive to heat and obviously upregulated under heat stress, as well as *TaCDPK2*/*5* to cold stress, *TaCDPK15* to drought stress, and *TaCDPK15*/*25* to sodium hydrosulfide (NaHS) stress (**Figure 5** and **Table 1**). Previous studies showed that the homologues of CDPKs played versatile functions in plants in response to different abiotic stresses. For instance, StCDPK32 (Zhu et al., 2021), ZmCPK11 (Borkiewicz et al., 2020), AtCPK12 (Zhang et al., 2018), OsCDPK21 (Asano et al., 2011), and AtCPK3 (Mehlmer et al., 2010) were all required for plant adaptation or response to salinity stress. In rice, the expression level of *OsCDPK13* was also increased in leaf sheath segments upon subjected to cold stress (Yang et al., 2003). On the contrary, ZmCPK1 was identified as a negative regulator in cold stress signaling (Weckwerth et al., 2015). CsCDPK20 and CsCDPK26 might act as a positive regulator in tea plant (*Camellia sinensis*) response to heat stress (Wang et al., 2018). In foxtail millet (*Setaria italica*), overexpression of SiCDPK24 in plants enhanced drought resistance and improved the survival rate under drought stress (Yu et al., 2018b). Moreover, overexpression of GmCDPK3 also improved plant tolerance to drought as well as salt stresses (Wang et al., 2019b). Besides these, OsCDPK1 also conferred drought tolerance in rice seedlings besides of its functions in seed development (Ho et al., 2013; Jiang et al., 2018). Based on the inducible expression profile of *TaCDPK2*, the high identification between TaCDPK2 and OsCDPK13 with 95.9%, we speculate that TaCDPK2 perhaps play the potential role in plant response to cold stress. In addition, although the induced expression profile (**Figures 3**, **7**) did not give a clear picture of the response of TaCDPK27 to cold stress, the more cold response-elements in the promoter of TaCDPK27 (**Figure S3**) and its high identification with ZmCPK1 (83.0% in **Table S5**) also suggesting its potential role in plant response to cold stress.

Thirdly, under biological stresses, the expression levels of different groups showed different responses (in **Figures 5**, **6B** and **Table 1**). For example, the members from the group I (*TaCDPK2*/*7*/*12*/*15*/*25*/*26*) were upregulated, but downregulated in the group II (*TaCDPK5*/*6*/*8*/*9*/*13*/*17*/*22*/*28*), and barely responded in the group III (*TaCDPK11*/*19*/*20*/*23*/*24*/*29*/*30*), under the treatment of *F. graminearum*, which is the main pathogen of FHB. While, *TaCDPK4*/*10*/*12*/*14*/*15*/*25* were all downregulated under *P. graminis* infection, which is the main pathogen of wheat stem rust. *TaCDPK5*/*10*/*15*/*25* were susceptive to *P. striiformis* (*Puccinia striiformis*, wheat stripe rust pathogen), as well as *TaCDPK4*/*10*/*12* to flg22 (pattern pathogenic elicitors from bacteria). These results suggested that different members with different genes and protein structures may also have the similar biological functions, such as TaCDPK15 and TaCDPK25 to *F. graminearum* as well as to *P. graminis* and *P. striiformis* in plants. On the other hand, the same members of TaCDPKs may show different biological functions in response to different biological stresses. For example, the expression level of *TaCDPK12* was upregulated under *F. graminearum* or flg22 stresses, but downregulated under *P. graminis* stress. Up to now, lots of CDPK homologs have been reported to be involved in plant immune processes. In *Arabidopsis*, AtCPK28, the homologs of TaCDPK6 (with the identification of 81.2%), as a negative regulator that continually buffers immune signaling by controlling the turnover of the plasma-membrane-associated cytoplasmic kinase BIK1, which is a rate limiting in pathogen-associated molecular patterns (PAMP)-triggered immunity (PTI) signaling (Monaghan et al., 2014). In addition, AtCPK5/CPK6 signaling pathways contribute to defense against *Botrytis cinerea* by promoting the biosynthesis of 4-methoxyindole-3-ylmethylglucosinolate and camalexin in plants (Yang et al., 2020b).

Finally, the induced expression profiles in **Figure 6A** indicated that TaCDPK1-5/7/12/16/19/22 were all sensitive to hormone MeJA, which are consistent with the results that most members of TaCDPKs harboring MeJA-responsive element (JARE) in their promoters (**Figure S3**). The results mentioned above suggesting that these TaCDPK family members perhaps widely involved in MeJA-mediated signaling pathways, which play important roles in plant growth, development, senescence, and in response to biotic and abiotic stresses (Shu et al., 2020; Ma et al., 2021; Raza et al., 2021; Wei et al., 2021). Whereas, TaCDPK2/3/7/8/12/15/16/19-21/25/26 were all sensitive to hormone BR as well as TaCDPK12/27 to SA and TaCDPK13 to ABA. Moreover, increasingly compelling evidences indicated that CDPKs involved in many hormone-mediated signaling in plants. In *Arabidopsis*, AtCPK6 the homologue of TaCDPK1 (identification 70.3%) was demonstrated that it functioned as a positive regulator in MeJA signaling in *Arabidopsis* guard cells as well as ABA-induced stomatal closure (Munemasa et al., 2011; Brandt et al., 2012). Another report showed that AtCPK12 negatively regulates abscisic acid signaling in seed germination and post-germination growth (Zhao et al., 2011). In addition, AtCPK29 was involved in the process of auxin efflux transport, polarity and auxin responses by specifically phosphorylating the target residues on the auxin efflux transporter (PIN) (Lee et al., 2021). In this paper, the expression level of TaCDPK13 in anther/flowering stage and its sensitivity to ABA (**Table 1**) were all consistent with our previous study, that TaCDPK13 played crucial roles in plant fertility, and drought tolerance (Hu et al., 2020a). Such results strongly support the idea that TaCDPK13 may be involved in drought response *via* ABA-dependent signal pathways.

### 4.3 The complicated interactions between TaCDPKs and TaNOXs perhaps play vital roles in plant development and response to stresses by regulating ROS production

**Figure 10:**
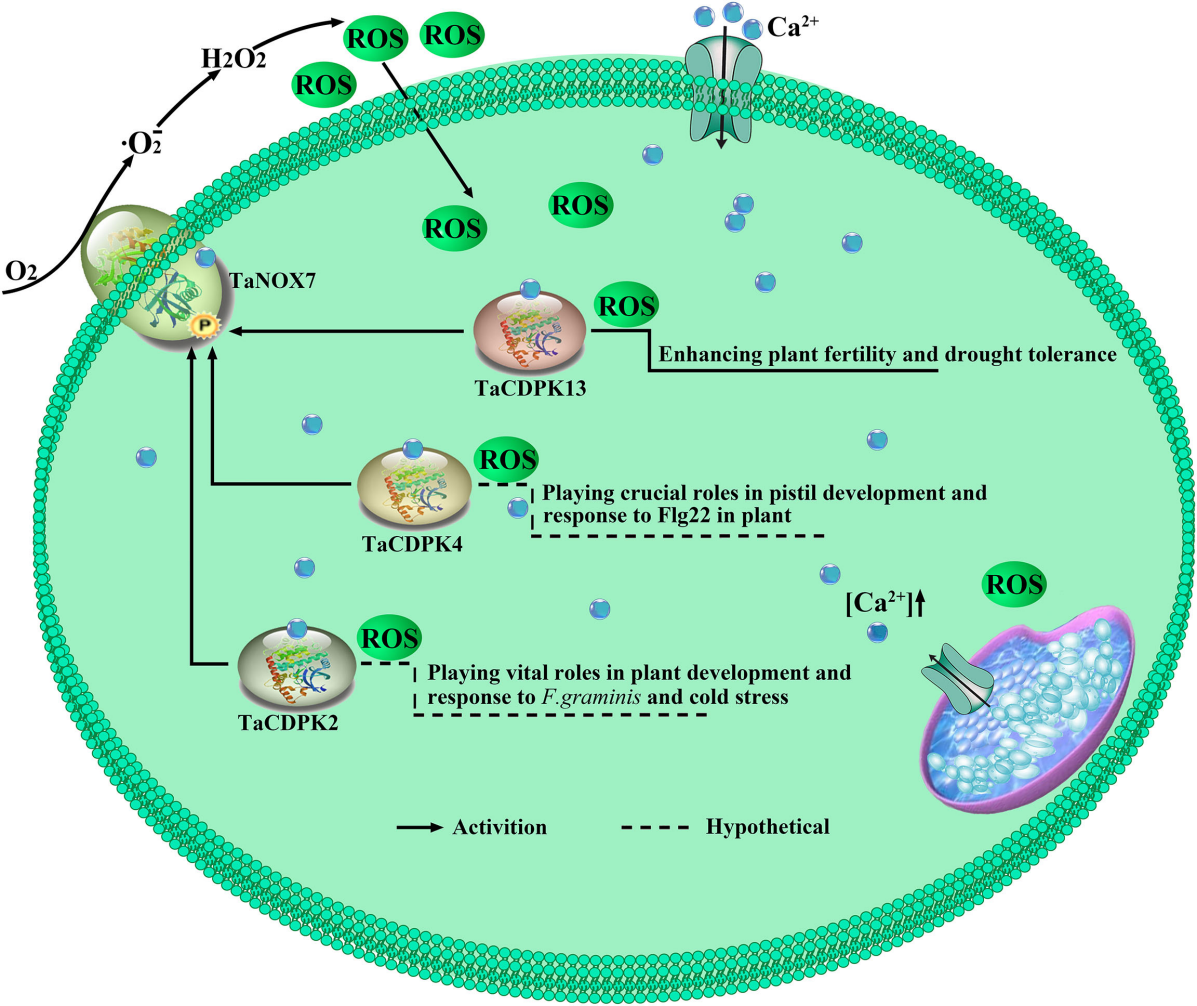
TaCDPK2/4/13-TaNOX7 interactions play crucial roles in plant by regulating ROS production. TaCDPK2/4 and TaCDPK13 (Hu et al., 2020a) can interact with and activate TaNOX7 for ROS production, which plays crucial roles in plant development.

It is well known that ROS and Ca^2+^ are universal and important intracellular signaling molecules, and their homeostasis play important roles in plant growth and development as well as response to biotic and abiotic stresses. More importantly, ROS and Ca^2+^, both as the signaling messengers, have complex cross-talks during signaling. For instance, Ca^2+^ binding can activate CDPKs to phosphorylate NADPH oxidases OsRbohB for ROS production (Kobayashi et al., 2007), which is necessary for Ca^2+^ influx, and then the induced ROS in turn may trigger Ca^2+^ efflux from intracellular Ca^2+^ stores *in vivo* (McAinsh MR et al., 1996; Pei et al., 2000). It should be added here that NADPH oxidases (NOXs), mostly known as respiratory burst oxidase homologs (RBOHs), are the key producers of ROS in plants (Hu et al., 2020b). Moreover, both TaCDPKs and TaNOXs all contain the EF_Hand, which are capable of calcium binding domains and thereby activate the protease activity of CDPKs and NOXs by cooperating with other particles (Hu et al., 2020b). Therefore, there must be complicated interactions between the members of CDPKs and NOXs in plants. Previously, we found that TaCDPK13 can interact with TaNOX7 for the plant fertility and drought tolerance (Hu et al., 2020a). Here, we found that the two others, TaCDPK2/4, can also directly interact with TaNOX7 and coexpression of the CDPKs with TaNOX7 enhanced ROS production (**Figures 7**, **8**). Intriguingly, increasing literatures reported that CDPKs-NOXs/RBOHs interactions played important roles in plants by regulating ROS homeostasis. For example, OsRboh mediated ROS production, which is induced by OsCDPK5/OsCDPK13, is essential for aerenchyma formation in rice roots (Yamauchi et al., 2017). In addition, StCDPK23 may participate in the wound healing of potato tubers by regulating StRBOHs for H_2_O_2_ production (Ma et al., 2022). AtCPK5 phosphorylates AtRbohD and enhances ROS production for defense responses and bacterial resistance (Dubiella et al., 2013); BnaCPK6L phosphorylates BnaRBOHD and increases the accumulation of ROS and HR-like cell death (Pan et al., 2019). More intriguingly, OsCPK12 promotes the tolerance of rice to salt stress by repressing the expression level of OsRbohI and reducing the accumulation of ROS (Asano et al., 2012; Boudsocq and Sheen, 2013). Therefore, CDPKs can regulate the activity of NOXs/RBOHs for regulating ROS homeostasis in plants, which plays diverse and vital functions in plants. Then, what are the biological significances of TaCDPK2/4-TaNOX7 interactions? All the expression patterns and analyses from **Figures S1/S2**, **4**–**6**, **9** and **Table 1** showed that TaCDPK2 was mainly expressed in young leaves, flag leaves and has significant response to *F.graminis* and cold stress; TaCDPK4 was mainly expressed in the pistils and has obvious response to Flagelin 22 (Flg22). In addition, our previous results showed that TaNOX7 was expressed in almost all the tissues of wheat and had high sensitivity to many stresses (Hu et al., 2018). TaCDPK13 can also interact with and activate TaNOX7 for ROS production, which can enhance plant fertility and drought tolerance (Hu et al., 2020a). Based on all the results, we concluded that, as shown in the model in **Figure 10**, TaCDPK2/4-TaNOX7 interactions mediated ROS homeostasis perhaps also plays crucial roles during the progress of vegetative and reproductive growth, seed development, and fertility in plants, respectively. In addition, they perhaps also play essential roles in plant response to biotic and abiotic stresses, such as cold, *F.graminis* stresses and so on.

In summary, wheat has multiple members of CDPKs with diverse but vital functions in plant growth, development regulation and stress responses. Every member of TaCDPKs has its specific expression pattern and function. Moreover, the synergistic or antagonistic interactions between TaCDPKs and TaNOXs are complicated and play important roles by regulating ROS level in plant, though the regulatory mechanism and biological significance of them are still under investigation. Therefore, the results obtained here have provided a valuable foundation for further exploring the functions and the signal pathways of CDPK superfamily members, especially the interactions between TaCDPKs and TaNOXs in wheat.

## Data availability statement

The original contributions presented in the study are included in the article/**Supplementary Material**. Further inquiries can be directed to the corresponding authors.

## Author contributions

K-M C, L-L L, and K-S M proposed the concept and content. C-H H, and B-B L wrote the manuscript. P C, H-Y S, and W-G X revised the manuscript. Y Z, Z-H Y and H-X W helped in the sample collection and experiment. All authors contributed to the article and approved the submitted version.
