# Supplementary material for: Identification of CDPKs involved in TaNOX7 mediated ROS production in wheat
Source: Front Plant Sci. 2023 Jan 23;13:1108622. doi: 10.3389/fpls.2022.1108622 (PMC9900008; doi:10.3389/fpls.2022.1108622)
Supplement: Supplementary file 1 [file DataSheet_1.pdf]

# *Supplementary Material*

## 1 Supplementary Data

All supplementary files are deposited to FigShare for permanent storage and receive a DOI.

## 2 Supplementary Figures and Tables

### 2.1 Supplementary Figures

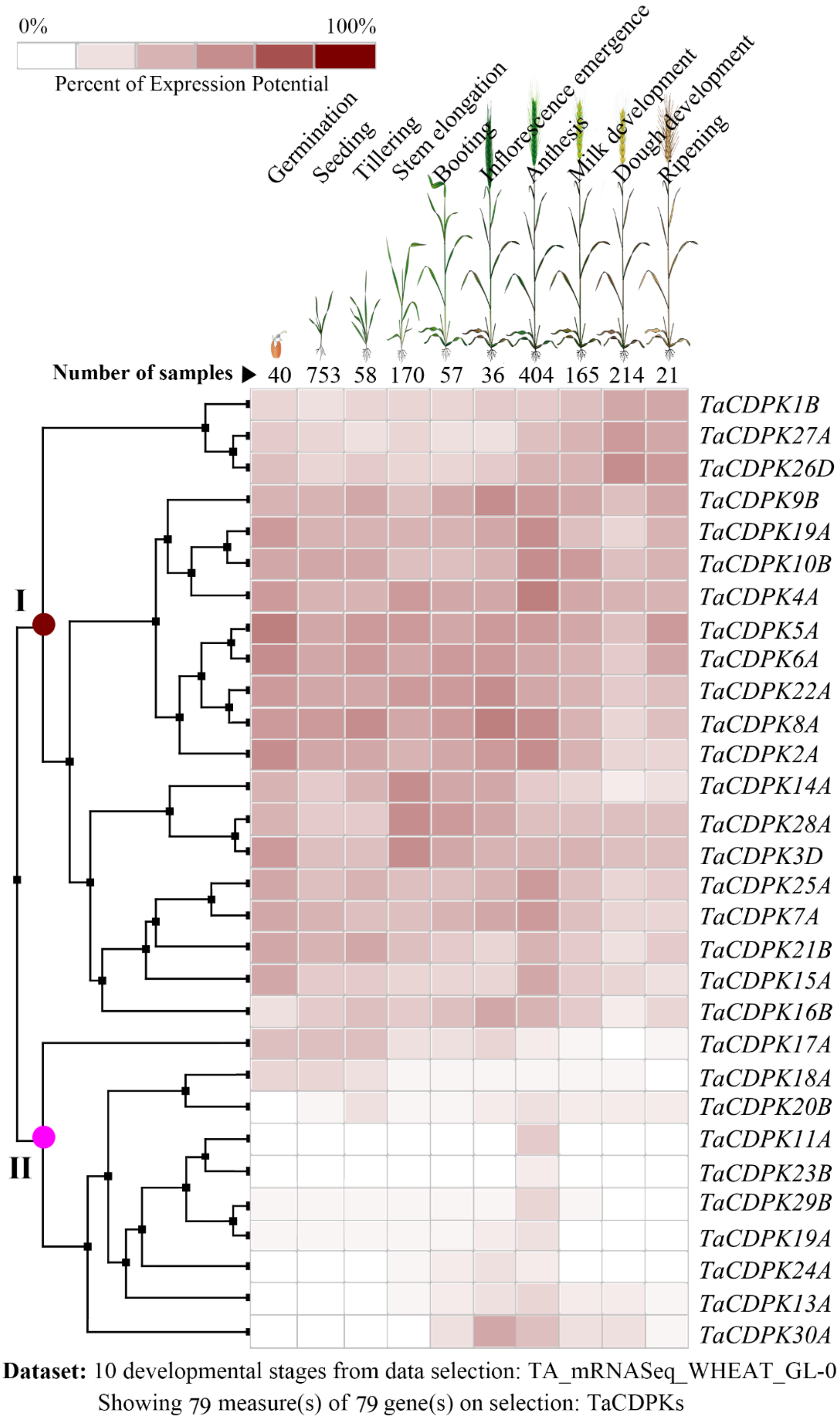


**Figure S1 Spatio-temporal expression patterns of TaCDPKs in wheat**

Expression patterns of CDPK and CRK genes in wheat at 10 different developmental stages were selected from the Ta_mRNASeq_WHEAT_GL-0 database in Genevestigator v3.

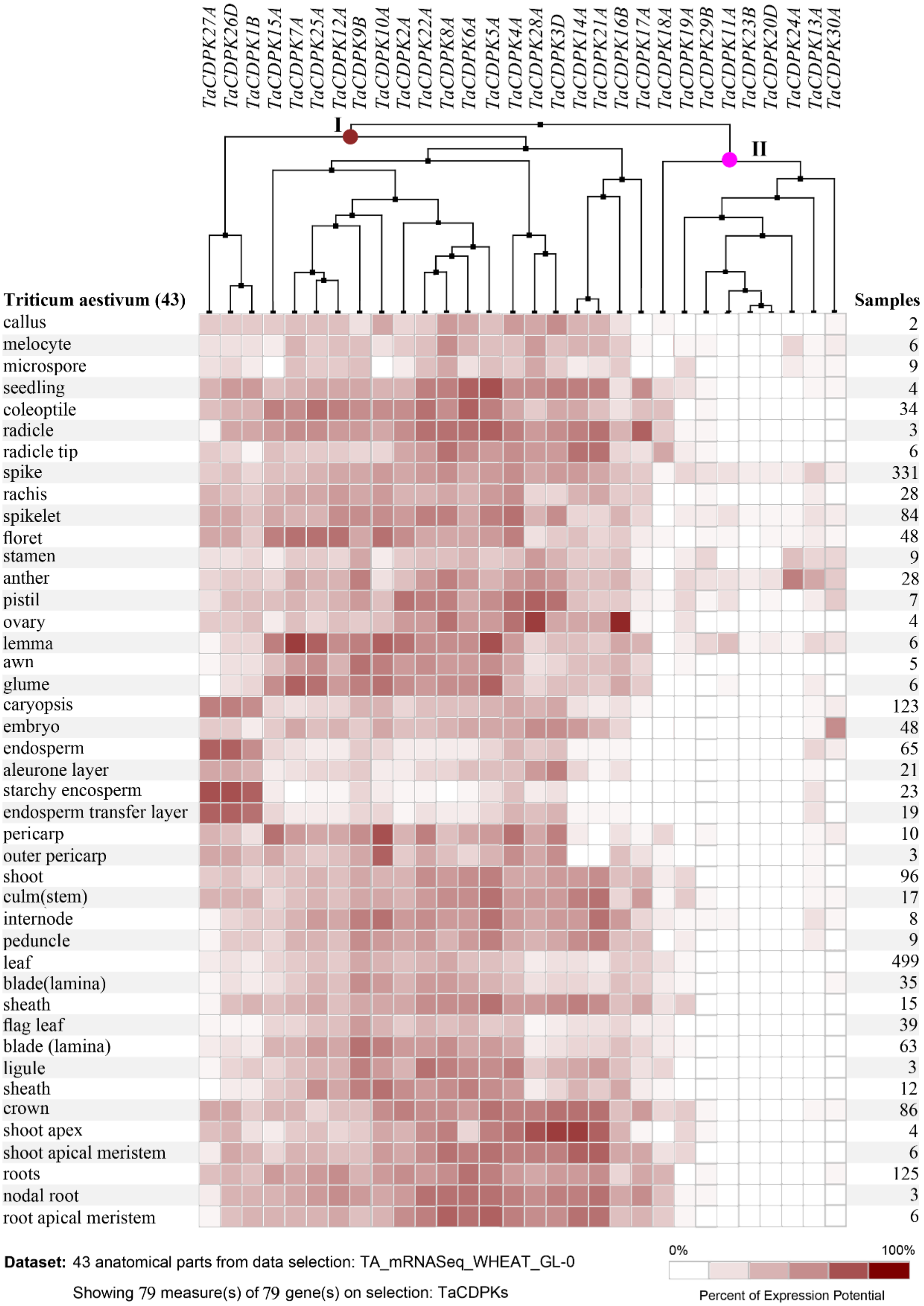


**Figure S2 The tissue-specific expression profiles of TaCDPKs in wheat.**

Expression patterns of CDPK genes in wheat at 43 different tissues were selected from Ta_mRNASeq_WHEAT_GL-0 database in Genevestigator v3.

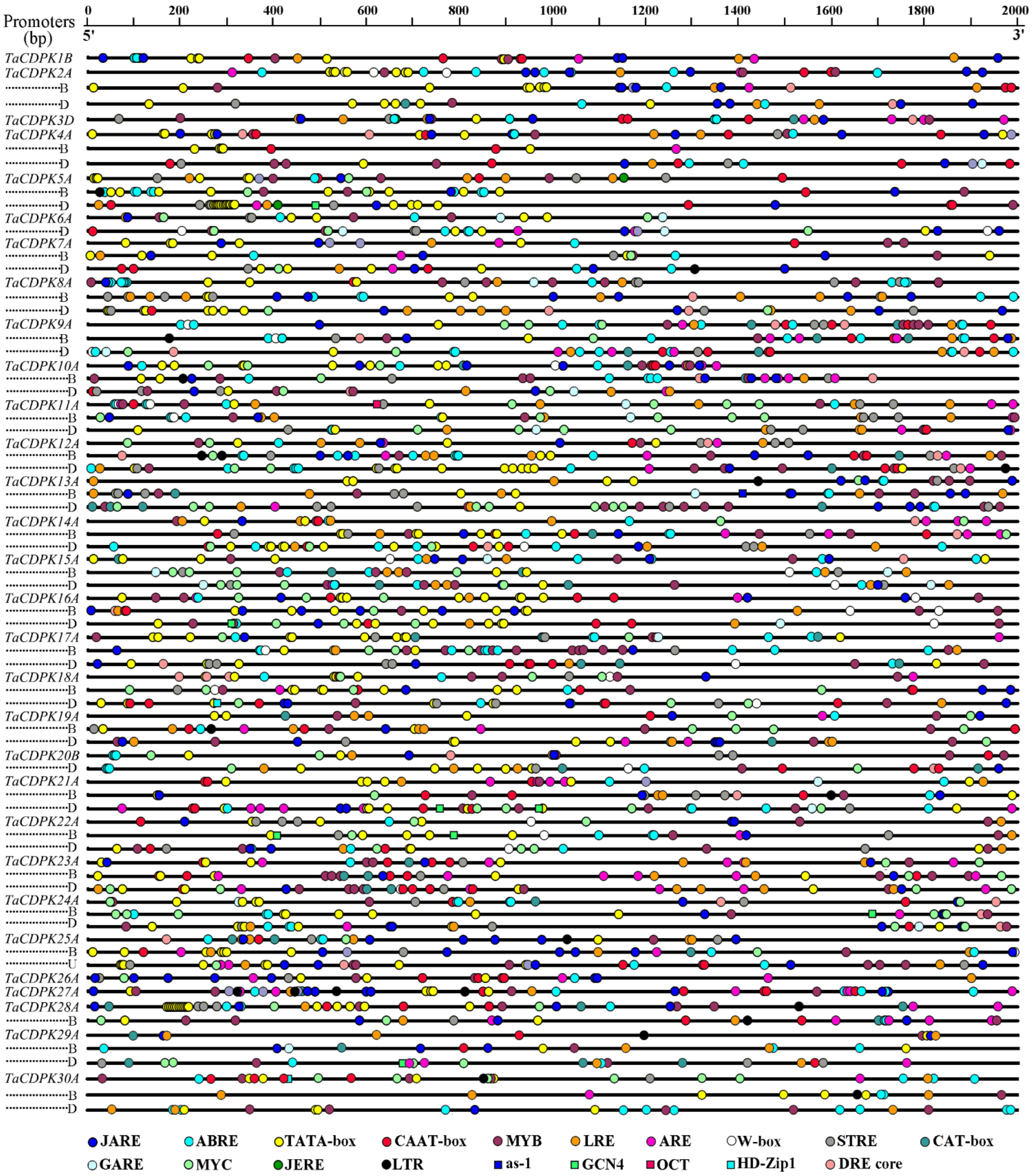


**Figure S3 The cis-elements in the sense strands of TaCDPK promoters.**

The cis-elements are as follows. Methl jasmonic acid (MeJA)-responsive element (JARE): TGACG motif and CGTCA motif; abscisic acid-responsive element (ABRE): ABRE, GC-motif, CAG-motif and AAGAA-motif; TATA-box: core promoter element around -30 bp of transcription start; CAAT-box: common cis-acting element in promoter and enhancer regions; MYB-responsive element (MRE): MYB binding site; light-responsive element (LRE): G-box, SPI, chs-Unit1 ml, GT1-motif, ACA-motif, GATA-motif, I-box, GATT-motif, AE-motif, and so on; aerobic responsive element

(ARE): ARE, CCAAT-box and GC-motif; W-box: WARK binding site; STRE: abiotic stress response element; CAT-box: cis-acting regulatory element related to meristem expression; gibberellin-responsive elemen (GARE), TATC-box, P-box, and GARE elements; Myc binding site (MYC); JERE: JA and elicitor responsive elements; LTR: low temperature responsiveness; as-1: salicylic acid (SA) responsiveness; GCN4: cis-regulatory element involved in endosperm expression; OCT: Meristem expression element; HD-Zip1: element involved in differentiation of the palisade mesophyll cells; DRE core: dehydration reaction element.

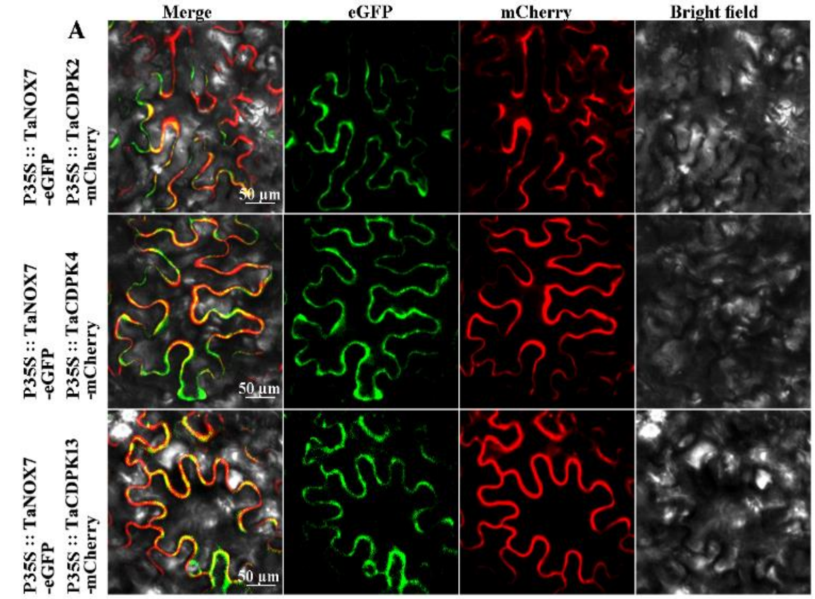


**Figure S4 Subcellular co-localization of TaCDPK2/4/13 and TaNOX7.**

## 2.2 Supplementary Tables

Table S1. The detailed information about the members of wheat *CDPKs* gene family

| Nomenclature | Gene names related to Chr. | Chr. | Gene ID | A.A | Loc | TMH/(AA) | MW/Da |
|---|---|---|---|---|---|---|---|
| *TaCDPK1* | *TaCDPK1B* | 4B | TraesCS4B02G321800.1 | 548 | Plas | 2(34.49201) | 67.45 |
| *TaCDPK2* | *TaCDPK2A* | 2A | TraesCS2A02G456100.1 | 558 | Plas/Mito | 0(0.27903) | 55.17 |
| | *TaCDPK2B* | 2B | TraesCS2B02G478100.1 | 559 | Plas/Mito | 0(0.27652) | 55.2 |
| | *TaCDPK2D* | 2D | TraesCS2D02G456400.1 | 532 | Plas/Mito | 0(0.28415) | 61.4 |
| *TaCDPK3* | *TaCDPK3D* | 3D | TraesCS3D02G345500.1 | 543 | Cyto | 0(1.90297) | 52.16 |
| *TaCDPK4* | *TaCDPK4A* | 4A | TraesCS4A02G206200.2 | 513 | Chlo | 0(1.07141) | 56.71 |
| | *TaCDPK4B* | 4B | TraesCS4B02G109800.2 | 513 | Chlo | 0(1.07141) | 56.72 |
| | *TaCDPK4D* | 4D | TraesCS4D02G107200.1 | 514 | Chlo | 0(1.06079) | 56.82 |
| *TaCDPK5* | *TaCDPK5A* | 2A | TraesCS2A02G223100.2 | 532 | Chlo | 0(7.20594) | 59.45 |
| | *TaCDPK5B* | 2B | TraesCS2B02G248700.2 | 532 | Chlo | 0(7.2061) | 59.42 |
| | *TaCDPK5D* | 2D | TraesCS2D02G229100.2 | 541 | Chlo | 0(11.25951) | 60.46 |
| *TaCDPK6* | *TaCDPK6A* | 2A | TraesCS2A02G239900.1 | 509 | Chlo/Plas | 0(0.01845) | 57.04 |
| | *TaCDPK6D* | 2D | TraesCS2D02G245700.1 | 509 | Chlo/Plas | 0/(0.01839) | 57.03 |
| *TaCDPK7* | *TaCDPK7A* | 2A | TraesCS2A02G199700.1 | 543 | Cyto | 0/(1.10096) | 60.98 |
| | *TaCDPK7B* | 2B | TraesCS2B02G227000.1 | 543 | Cyto | 0(1.10154) | 60.96 |
| | *TaCDPK7D* | 2D | TraesCS2D02G207400.1 | 543 | Cyto | 0(1.10154) | 60.97 |
| *TaCDPK8* | *TaCDPK8A* | 3A | TraesCS3A02G224900.1 | 518 | Mito | 0(6.98967) | 58.42 |
| | *TaCDPK8B* | 3B | TraesCS3B02G254500.1 | 518 | Mito | 0(6.98967) | 58.42 |
| | *TaCDPK8D* | 3D | TraesCS3D02G228700.1 | 518 | Mito | 0(6.98967) | 58.39 |
| *TaCDPK9* | *TaCDPK9A* | 2A | TraesCS2A02G248200.3 | 566 | Chlo/ Plas | 0(0.078) | 62.05 |
| | *TaCDPK9B* | 2B | TraesCS2B02G269200.2 | 566 | Chlo/ Plas | 0(0.078) | 62.06 |
| | *TaCDPK9D* | 2D | TraesCS2D02G249900.2 | 562 | Chlo/ Plas | 0(0.08176) | 61.74 |
| *TaCDPK10* | *TaCDPK10A* | 1A | TraesCS1A02G424200.1 | 460 | Plas/ Cysk | 0(5.1896) | 52.2 |
| | *TaCDPK10B* | 1B | TraesCS1B02G456700.1 | 460 | Plas/ Cysk | 0(3.52519) | 52.18 |
| | *TaCDPK10D* | 1D | TraesCS1D02G433400.1 | 460 | Plas/ Cysk | 0(5.18667) | 52.22 |
| *TaCDPK11* | *TaCDPK11A* | 5A | TraesCS5A02G118200.1 | 553 | Chlo | 0(4.0684) | 62.11 |
| | *TaCDPK11B* | 5B | TraesCS5B02G112900.1 | 550 | Chlo | 0(2.73504) | 61.6 |
| | *TaCDPK11D* | 5D | TraesCS5D02G119100.2 | 595 | Chlo | 0(2.99793) | 66.08 |
| *TaCDPK12* | *TaCDPK12A* | 5A | TraesCS5A02G463100.2 | 534 | Mito | 0(0.87099) | 59.84 |
| | *TaCDPK12B* | 5B | TraesCS5B02G474500.1 | 535 | ER | 0(0.86141) | 59.94 |
| | *TaCDPK12D* | 5D | TraesCS5D02G475900.1 | 534 | Mito | 0(0.87395) | 59.82 |
| *TaCDPK13* | *TaCDPK13A* | 3A | TraesCS3A02G334200.1 | 518 | Plas | 0(3.49213) | 57.06 |
| | *TaCDPK13B* | 3B | TraesCS3B02G365200.1 | 518 | Plas | 0(3.49157) | 57.23 |
| | *TaCDPK13D* | 3D | TraesCS3D02G327700.3 | 520 | Plas | 0(3.46166) | 57.16 |
| *TaCDPK14* | *TaCDPK14A* | 1A | TraesCS1A02G292200.1 | 551 | All | 0(1.5661) | 60.97 |
| | *TaCDPK14B* | 1B | TraesCS1B02G301600.1 | 549 | All | 0(1.54649) | 60.91 |
| | *TaCDPK14D* | 1D | TraesCS1D02G290700.1 | 549 | All | 0(1.54701) | 60.9 |
| *TaCDPK15* | *TaCDPK15A* | 5A | TraesCS5A02G426500.1 | 627 | Chlo | 0(0.43291) | 68.46 |

| | | | | | | | |
|---|---|---|---|---|---|---|---|
| | *TaCDPK15B* | 5B | TraesCS5B02G428400.2 | 627 | Chlo | 0(0.43291) | 68.48 |
| | *TaCDPK15D* | 5D | TraesCS5D02G434500.2 | 624 | Chlo | 0(0.44466) | 68.23 |
| *TaCDPK16* | *TaCDPK16A* | 4A | TraesCS4A02G187900.1 | 532 | Cyto | 0(6.67024) | 58.78 |
| | *TaCDPK16B* | 4B | TraesCS4B02G130300.1 | 532 | Cyto | 0(7.58295) | 58.79 |
| | *TaCDPK16D* | 4D | TraesCS4D02G125500.1 | 532 | Cyto | 0(7.16015) | 58.78 |
| *TaCDPK17* | *TaCDPK17A* | 2A | TraesCS2A02G407200.1 | 539 | Cyto | 0(1.58072) | 60.1 |
| | *TaCDPK17B* | 2B | TraesCS2B02G424900.1 | 537 | Cyto | 0(1.6147) | 60.08 |
| | *TaCDPK17D* | 2D | TraesCS2D02G404200.1 | 534 | Cyto | 0(1.66722) | 59.62 |
| *TaCDPK18* | *TaCDPK18A* | 4A | TraesCS4A02G283400.1 | 569 | Chlo | 0(1.19775) | 63.19 |
| | *TaCDPK18B* | 4B | TraesCS4B02G030100.1 | 600 | Chlo | 0(1.13441) | 66.81 |
| | *TaCDPK18D* | 4D | TraesCS4D02G027600.1 | 570 | Chlo | 0(0.8194) | 63.22 |
| *TaCDPK19* | *TaCDPK19A* | 5A | TraesCS5A02G426800.2 | 610 | Chlo | 0(0.12202) | 67.11 |
| | *TaCDPK19B* | 5B | TraesCS5B02G428700.1 | 586 | Chlo | 0(0.11657) | 64.21 |
| | *TaCDPK19D* | 5D | TraesCS5D02G434800.2 | 610 | Chlo | 0(0.12139) | 67.13 |
| *TaCDPK20* | *TaCDPK20B* | 5B | TraesCS5B02G109300.1 | 624 | Mito | 0(0.7491) | 68.91 |
| | *TaCDPK20D* | 5D | TraesCS5D02G124000.1 | 624 | Mito | 0(2.09503) | 69.02 |
| *TaCDPK21* | *TaCDPK21A* | 6A | TraesCS6A02G261200.1 | 550 | Plas/ Mito | 0(1.62419) | 60.66 |
| | *TaCDPK21B* | 6B | TraesCS6B02G288600.1 | 548 | Plas/ Mito | 0(1.65799) | 60.47 |
| | *TaCDPK21D* | 6D | TraesCS6D02G242000.2 | 548 | Plas/ Mito | 0(1.65799) | 60.52 |
| *TaCDPK22* | *TaCDPK22A* | 5A | TraesCS5A02G107900.1 | 564 | Plas | 0(5.81767) | 62.3 |
| | *TaCDPK22B* | 5B | TraesCS5B02G115400.1 | 529 | Plas | 0(0.54357) | 58.3 |
| | *TaCDPK22D* | 5D | TraesCS5D02G122800.1 | 529 | Plas | 0(0.50599) | 58.27 |
| *TaCDPK23* | *TaCDPK23A* | 5A | TraesCS5A02G138300.1 | 541 | Pero | 0(1.76203) | 59.62 |
| | *TaCDPK23B* | 5B | TraesCS5B02G137600.1 | 539 | Pero | 0(1.96494) | 59.41 |
| | *TaCDPK23D* | 5D | TraesCS5D02G153800.1 | 541 | Pero | 0(1.76268) | 59.7 |
| *TaCDPK24* | *TaCDPK24A* | 1A | TraesCS1A02G309400.2 | 517 | Chlo | 0(13.87427) | 57.39 |
| | *TaCDPK24B* | 1B | TraesCS1B02G320400.1 | 517 | Chlo | 0(12.15675) | 57.43 |
| | *TaCDPK24D* | 1D | TraesCS1D02G308800.1 | 517 | Mito/Chlo | 0(9.36491) | 57.33 |
| *TaCDPK25* | *TaCDPK25A* | 6A | TraesCS6A02G089400.2 | 511 | Cyto | 0(0.02115) | 57.65 |
| | *TaCDPK25B* | 6B | TraesCS6B02G111800.1 | 511 | Cyto | 0(0.02115) | 57.65 |
| | *TaCDPK25Un* | Un | TraesCSU02G016900.1 | 512 | Cyto | 0(0.02107) | 57.72 |
| *TaCDPK26* | *TaCDPK26D* | 4D | TraesCS4D02G318400.1 | 548 | Cyto | 0(0.73061) | 61.33 |
| *TaCDPK27* | *TaCDPK27A* | 5A | TraesCS5A02G490200.1 | 548 | Cyto | 0(0.73394) | 61.38 |
| *TaCDPK28* | *TaCDPK28A* | 3A | TraesCS3A02G351300.1 | 547 | Cyto | 0(1.59547) | 60.97 |
| | *TaCDPK28B* | 3B | TraesCS3B02G383800.1 | 542 | Cyto | 0(1.63891) | 60.75 |
| *TaCDPK29* | *TaCDPK29A* | 5A | TraesCS5A02G298500.1 | 548 | Nucl | 0(0.00000) | 61.22 |
| | *TaCDPK29B* | 5B | TraesCS5B02G297700.1 | 552 | Nucl | 0(0.00000) | 61.79 |
| | *TaCDPK29D* | 5D | TraesCS5D02G305200.1 | 552 | Nucl | 0(0.00000) | 61.79 |
| *TaCDPK30* | *TaCDPK30A* | 7A | TraesCS7A02G267000.1 | 583 | Nucl | 0(0.00000) | 64.09 |
| | *TaCDPK30B* | 7B | TraesCS7B02G165200.1 | 577 | Nucl | 0(0.00000) | 63.54 |
| | *TaCDPK30D* | 7D | TraesCS7D02G267700.1 | 584 | Nucl | 0(0.00000) | 64.23 |

The data source of wheat *TaCDPKs* from IWGSC (http://www.wheatgenome.org/), e!EnsemblPlants (http://plants.ensembl.org/index.html), ExPASy (http://web.expasy.org/protparam/), Plant-mPLoc (http://www.csbio.sjtu.edu.cn/bioinf/plant-multi/), DeepTMHMM (https://dtu.biolib.com/DeepTMHMM),

GenScript (https://www.genscript.com/tools/wolf-psort). Chr.: chromosome; A.A.: number of amino acids; Loc: the subcellular localization; TMH (AA), also known as TMHelix (AA), represents the number of predicted transmembrane helices and the number of amino acids in the membrane; MW: molecular weight; Plas: plasma membrane; Cysk: cytoskeleton; Mito: mitochondrion; Pero: peroxisome; Chlo: chloroplast; Nucl: Nucleus; Cyto: cytoplas.

Table S2. The 132 homologous gene pairs in CDPK family

| | | | | | | | | | |
|---|---|---|---|---|---|---|---|---|---|
| 1 | 2 | 3 | 4 | 5 | 6 | 7 | 8 | 9 | 10 |
| *TaCDPK14A*<br>*TaCDPK14B* | *TaCDPK24A*<br>*TaCDPK24B* | *TaCDPK10A*<br>*TaCDPK10B* | *TaCDPK14A*<br>*TaCDPK14D* | *TaCDPK14B*<br>*TaCDPK14D* | *TaCDPK24A*<br>*TaCDPK24D* | *TaCDPK24B*<br>*TaCDPK24D* | *TaCDPK10A*<br>*TaCDPK10D* | *TaCDPK10B*<br>*TaCDPK10D* | *TaCDPK7A*<br>*TaCDPK7B* |
| 11 | 12 | 13 | 14 | 15 | 16 | 17 | 18 | 19 | 20 |
| *TaCDPK5A*<br>*TaCDPK5B* | *TaCDPK9A*<br>*TaCDPK9B* | *TaCDPK17A*<br>*TaCDPK17B* | *TaCDPK2A*<br>*TaCDPK2B* | *TaCDPK7A*<br>*TaCDPK7D* | *TaCDPK7B*<br>*TaCDPK7D* | *TaCDPK5A*<br>*TaCDPK5D* | *TaCDPK5B*<br>*TaCDPK5D* | *TaCDPK9A*<br>*TaCDPK9D* | *TaCDPK9B*<br>*TaCDPK9D* |
| 21 | 22 | 23 | 24 | 25 | 26 | 27 | 28 | 29 | 30 |
| *TaCDPK17A*<br>*TaCDPK17D* | *TaCDPK17B*<br>*TaCDPK17D* | *TaCDPK2A*<br>*TaCDPK2D* | *TaCDPK2B*<br>*TaCDPK2D* | *TaCDPK10A*<br>*TaCDPK8A* | *TaCDPK10B*<br>*TaCDPK8A* | *TaCDPK10D*<br>*TaCDPK8A* | *TaCDPK24A*<br>*TaCDPK13A* | *TaCDPK24B*<br>*TaCDPK13A* | *TaCDPK24D*<br>*TaCDPK13A* |
| 31 | 32 | 33 | 34 | 35 | 36 | 37 | 38 | 39 | 40 |
| *TaCDPK14A*<br>*TaCDPK28A* | *TaCDPK14B*<br>*TaCDPK28A* | *TaCDPK14D*<br>*TaCDPK28A* | *TaCDPK10A*<br>*TaCDPK8B* | *TaCDPK10B*<br>*TaCDPK8B* | *TaCDPK10D*<br>*TaCDPK8B* | *TaCDPK8A*<br>*TaCDPK8B* | *TaCDPK24A*<br>*TaCDPK13B* | *TaCDPK24B*<br>*TaCDPK13B* | *TaCDPK24D*<br>*TaCDPK13B* |
| 41 | 42 | 43 | 44 | 45 | 46 | 47 | 48 | 49 | 50 |
| *TaCDPK13A*<br>*TaCDPK13B* | *TaCDPK14A*<br>*TaCDPK28B* | *TaCDPK14B*<br>*TaCDPK28B* | *TaCDPK14D*<br>*TaCDPK28B* | *TaCDPK28A*<br>*TaCDPK28B* | *TaCDPK10A*<br>*TaCDPK8D* | *TaCDPK10B*<br>*TaCDPK8D* | *TaCDPK10D*<br>*TaCDPK8D* | *TaCDPK8A*<br>*TaCDPK8D* | *TaCDPK8B*<br>*TaCDPK8D* |
| 51 | 52 | 53 | 54 | 55 | 56 | 57 | 58 | 59 | 60 |
| *TaCDPK24A*<br>*TaCDPK13D* | *TaCDPK24B*<br>*TaCDPK13D* | *TaCDPK24D*<br>*TaCDPK13D* | *TaCDPK13A*<br>*TaCDPK13D* | *TaCDPK13B*<br>*TaCDPK13D* | *TaCDPK14A*<br>*TaCDPK3D* | *TaCDPK14B*<br>*TaCDPK3D* | *TaCDPK14D*<br>*TaCDPK3D* | *TaCDPK28A*<br>*TaCDPK3D* | *TaCDPK28B*<br>*TaCDPK3D* |
| 61 | 62 | 63 | 64 | 65 | 66 | 67 | 68 | 69 | 70 |
| *TaCDPK18A*<br>*TaCDPK18B* | *TaCDPK4A*<br>*TaCDPK4B* | *TaCDPK16A*<br>*TaCDPK16B* | *TaCDPK18A*<br>*TaCDPK18D* | *TaCDPK18B*<br>*TaCDPK18D* | *TaCDPK4A*<br>*TaCDPK4D* | *TaCDPK4B*<br>*TaCDPK4D* | *TaCDPK24A*<br>*TaCDPK16D* | *TaCDPK13D*<br>*TaCDPK16D* | *TaCDPK16A*<br>*TaCDPK16D* |
| 71 | 72 | 73 | 74 | 75 | 76 | 77 | 78 | 79 | 80 |
| *TaCDPK16B*<br>*TaCDPK16D* | *TaCDPK1B*<br>*TaCDPK26D* | *TaCDPK24A*<br>*TaCDPK23A* | *TaCDPK13A*<br>*TaCDPK23A* | *TaCDPK13D*<br>*TaCDPK23A* | *TaCDPK1B*<br>*TaCDPK27A* | *TaCDPK26D*<br>*TaCDPK27A* | *TaCDPK22A*<br>*TaCDPK20B* | *TaCDPK11A*<br>*TaCDPK11B* | *TaCDPK4A*<br>*TaCDPK22B* |
| 81 | 82 | 83 | 84 | 85 | 86 | 87 | 88 | 89 | 90 |
| *TaCDPK4B*<br>*TaCDPK22B* | *TaCDPK4D*<br>*TaCDPK22B* | *TaCDPK22A*<br>*TaCDPK22B* | *TaCDPK24A*<br>*TaCDPK23B* | *TaCDPK24D*<br>*TaCDPK23B* | *TaCDPK13A*<br>*TaCDPK23B* | *TaCDPK13D*<br>*TaCDPK23B* | *TaCDPK16A*<br>*TaCDPK23B* | *TaCDPK16B*<br>*TaCDPK23B* | *TaCDPK16D*<br>*TaCDPK23B* |
| 91 | 92 | 93 | 94 | 95 | 96 | 97 | 98 | 99 | 100 |

| | | | | | | | | | |
|---|---|---|---|---|---|---|---|---|---|
| *TaCDPK29A*<br>*TaCDPK29B* | *TaCDPK15A*<br>*TaCDPK15B* | *TaCDPK19A*<br>*TaCDPK19B* | *TaCDPK12A*<br>*TaCDPK12B* | *TaCDPK11B*<br>*TaCDPK11D* | *TaCDPK20B*<br>*TaCDPK22D* | *TaCDPK22B*<br>*TaCDPK22D* | *TaCDPK24A*<br>*TaCDPK23D* | *TaCDPK13A*<br>*TaCDPK23D* | *TaCDPK13D*<br>*TaCDPK23D* |
| 101 | 102 | 103 | 104 | 105 | 106 | 107 | 108 | 109 | 110 |
| *TaCDPK16B*<br>*TaCDPK23D* | *TaCDPK23A*<br>*TaCDPK23D* | *TaCDPK23B*<br>*TaCDPK23D* | *TaCDPK29A*<br>*TaCDPK29D* | *TaCDPK29B*<br>*TaCDPK29D* | *TaCDPK15A*<br>*TaCDPK15D* | *TaCDPK15B*<br>*TaCDPK15D* | *TaCDPK12A*<br>*TaCDPK12D* | *TaCDPK12B*<br>*TaCDPK12D* | *TaCDPK2A*<br>*TaCDPK21A* |
| 111 | 112 | 113 | 114 | 115 | 116 | 117 | 118 | 119 | 120 |
| *TaCDPK2B*<br>*TaCDPK21A* | *TaCDPK2D*<br>*TaCDPK21A* | *TaCDPK25A*<br>*TaCDPK25B* | *TaCDPK2A*<br>*TaCDPK21B* | *TaCDPK2B*<br>*TaCDPK21B* | *TaCDPK2D*<br>*TaCDPK21B* | *TaCDPK21A*<br>*TaCDPK21B* | *TaCDPK2A*<br>*TaCDPK21D* | *TaCDPK2B*<br>*TaCDPK21D* | *TaCDPK2D*<br>*TaCDPK21D* |
| 121 | 122 | 123 | 124 | 125 | 126 | 127 | 128 | 129 | 130 |
| *TaCDPK21A*<br>*TaCDPK21D* | *TaCDPK21B*<br>*TaCDPK21D* | *TaCDPK29A*<br>*TaCDPK30B* | *TaCDPK29B*<br>*TaCDPK30B* | *TaCDPK29D*<br>*TaCDPK30B* | *TaCDPK30A*<br>*TaCDPK30B* | *TaCRK1A*<br>*TaCRK3D* | *TaCDPK29A*<br>*TaCDPK30D* | *TaCDPK29B*<br>*TaCDPK30D* | *TaCDPK29D*<br>*TaCDPK30D* |
| 131 | 132 | | | | | | | | |
| *TaCDPK30A*<br>*TaCDPK30D* | *TaCDPK30B*<br>*TaCDPK30D* | | | | | | | | |

Table S3. The gene IDs used software TBtools for analyzing the signal network relationships between the members of CDPK and NOX family in wheat

| Gene Name | Gene ID from the first generation sequencing | Gene ID from the third generation sequencing |
|---|---|---|
| *TaNOX1-1AL* | Traes_1AL_F636EC6F4 | TraesCS1A02G290700 |
| *TaNOX1-1BL* | Traes_1BL_20258AC56 | TraesCS1B02G300000 |
| *TaNOX1-1DL* | Traes_1DL_F4590DE82 | TraesCS1D02G289300 |
| *TaNOX2-1AL* | Traes_1AL_7EAFBE83A | TraesCS1A02G347700 |
| *TaNOX2-1DL* | Traes_1DL_90D078159 | TraesCS1D02G350600 |
| *TaNOX3-1BL* | Traes_1BL_7237DD9CD | TraesCS1B02G295200 |
| *TaNOX3-1DL* | Traes_1DL_7DA75D562 | TraesCS1D02G284900 |
| *TaNOX4-1DL* | Traes_1DL_C8A434071 | TraesCS1D02G284800 |
| *TaNOX5-3AL* | Traes_3AL_D03D5E3A0 | TraesCS3A02G354200 |
| *TaNOX5-3BL* | Traes_3B_FF5B3D6BB | TraesCS3B02G386600 |
| *TaNOX5-3DL* | Traes_3DL_19E13300B | TraesCS3D02G347900 |
| *TaNOX6-3AL* | Traes_3AL_FE33443AF | TraesCS3A02G280200 |
| *TaNOX6-3BL* | Traes_3B_5D8EA5CD1 | TraesCS3B02G314000 |
| *TaNOX6-3DL* | Traes_3DL_927AB8E99 | TraesCS3D02G279900 |
| *TaNOX7-3AS* | Traes_3AS_6E3C01A0F | TraesCS3A02G182900 |
| *TaNOX7-3BS* | Traes_3B_50DC948CA | TraesCS3B02G212900 |
| *TaNOX7-3DS* | Traes_3DS_03549D097 | TraesCS3D02G187300 |
| *TaNOX8-4BL* | Traes_4BL_A632599AF | TraesCS4B02G358800 |
| *TaNOX8-4DL* | Traes_4DL_9CC7EB9A1 | TraesCS4D02G352200 |
| *TaNOX9-4DL* | Traes_4DL_76F6959C5 | TraesCS4D02G324800 |
| *TaNOX10-5AL* | Traes_5AL_221649956 | TraesCS5A02G301700 |
| *TaNOX10-5BL* | Traes_5BL_CF34FCA90 | TraesCS5B02G299000 |
| *TaNOX11-5AL* | Traes_5AL_A35B74583 | TraesCS5A02G499900 |
| *TaNOX12-5AL* | Traes_5AL_12FDD7791 | TraesCS5A02G211800 |
| *TaNOX12-5BL* | Traes_5BL_686C04088 | TraesCS5B02G212100 |
| *TaNOX12-5DL* | Traes_5DL_51E9FC7F9 | TraesCS5D02G222100 |
| *TaNOX13-5AL* | Traes_5AL_68844CE9B | TraesCS5A02G527600 |
| *TaNOX14-5AS* | Traes_5AS_BF8B4ECE0 | TraesCS5A02G093600 |
| *TaNOX14-5BS* | Traes_5BS_BCC1B9791 | TraesCS5B02G099700 |
| *TaNOX14-5DS* | Traes_5DS_2809B6F77 | TraesCS5D02G105900 |
| *TaNOX15-6AS* | Traes_6AS_DE7524DF1 | TraesCS6A02G180600 |
| *TaNOX15-6BS* | Traes_6BS_08817BB20 | TraesCS6B02G205000 |
| *TaCDPK1-4BL* | Traes_4BL_46AF42528 | TraesCS4B02G321800 |
| *TaCDPK2-2AL* | Traes_2AL_7AAD9B6EF | TraesCS2A02G456100 |
| *TaCDPK2-2BL* | Traes_2BL_2141AFC9E | TraesCS2B02G478100 |
| *TaCDPK2-2DL* | Traes_XX_7E4C4EA93 | TraesCS2D02G456400 |
| *TaCDPK3-3DL* | Traes_3DL_D033F3219 | TraesCS3D02G345500 |
| *TaCDPK4-4AL* | Traes_4AL_D787CF1CC | TraesCS4A02G206200 |

| | | |
|---|---|---|
| *TaCDPK4-4BS* | Traes_4BS_9E2145C02 | TraesCS4B02G109800 |
| *TaCDPK4-4DS* | Traes_4DS_1E47439C6 | TraesCS4D02G107200 |
| *TaCDPK5-2AS* | Traes_2AS_FACC395EF | TraesCS2A02G223100 |
| *TaCDPK5-2BS* | Traes_2BS_6B6C8A542 | TraesCS2B02G248700 |
| *TaCDPK5-2DS* | Traes_2DS_E10233A82 | TraesCS2D02G229100 |
| *TaCDPK6-2A* | Traes_XX_3C1D2B511 | TraesCS2A02G239900 |
| *TaCDPK7-2AS* | Traes_2AS_6DA49285E | TraesCS2A02G199700 |
| *TaCDPK7-2BS* | Traes_2BS_4F489C2B9 | TraesCS2B02G227000 |
| *TaCDPK7-2DS* | Traes_2DS_153405778 | TraesCS2D02G207400 |
| *TaCDPK8-3AL* | Traes_3AL_F28856AD8 | TraesCS3A02G224900 |
| *TaCDPK8-3BL* | Traes_3B_C0F0C4EE3 | TraesCS3B02G254500 |
| *TaCDPK8-3DL* | Traes_3DL_17C29322D | TraesCS3D02G228700 |
| *TaCDPK9-2AS* | Traes_2AS_AFE52A9F8 | TraesCS2A02G248200 |
| *TaCDPK9-2BL* | Traes_2BL_4DA0C4B30 | TraesCS2B02G269200 |
| *TaCDPK9-2DL* | Traes_2DL_82900E142 | TraesCS2D02G249900 |
| *TaCDPK10-1AL* | Traes_1AL_3FDB6FDB4 | TraesCS1A02G424200 |
| *TaCDPK10-1BL* | Traes_1BL_B13C6589F | TraesCS1B02G456700 |
| *TaCDPK10-1DL* | Traes_1DL_90E3FC8F5 | TraesCS1D02G433400 |
| *TaCDPK11-5AS* | Traes_5AS_9A8A9187C | TraesCS5A02G118200 |
| *TaCDPK11-5BS* | Traes_5BS_DA5941467 | TraesCS5B02G112900 |
| *TaCDPK11-5DS* | Traes_5DS_8C4EC71C0 | TraesCS5D02G119100 |
| *TaCDPK12-5AL* | Traes_5AL_DEDF36AD2 | TraesCS5A02G463100 |
| *TaCDPK12-5BL* | Traes_5BL_35A6B4387 | TraesCS5B02G474500 |
| *TaCDPK12-5DL* | Traes_5DL_ADFFAE33D | TraesCS5D02G475900 |
| *TaCDPK13-3AL* | Traes_3AL_7A685D3E8 | TraesCS3A02G334200 |
| *TaCDPK13-3BL* | Traes_3B_A930AA6E9 | TraesCS3B02G365200 |
| *TaCDPK13-3DL* | Traes_3DL_82E77F57E | TraesCS3D02G327700 |
| *TaCDPK14-1AL* | Traes_1AL_C49C8F4AE | TraesCS1A02G292200 |
| *TaCDPK14-1BL* | Traes_1BL_4B5CD80AD | TraesCS1B02G301600 |
| *TaCDPK14-1DL* | Traes_1DL_19FE03F27 | TraesCS1D02G290700 |
| *TaCDPK15-5AL* | Traes_5AL_CF6660D45 | TraesCS5A02G426500 |
| *TaCDPK15-5DL* | Traes_5DL_321243AA8 | TraesCS5D02G434500 |
| *TaCDPK16-4AL* | Traes_4AL_E0E01F494 | TraesCS4A02G187900 |
| *TaCDPK16-4BS* | Traes_4BS_D7E5E888B | TraesCS4B02G130300 |
| *TaCDPK16-4DS* | Traes_4DS_8102D96DE1 | TraesCS4D02G125500 |
| *TaCDPK17-2AL* | Traes_2AL_4D3642D87 | TraesCS2A02G407200 |
| *TaCDPK17-2BL* | Traes_2BL_4265025D3 | TraesCS2B02G424900 |
| *TaCDPK17-2DL* | Traes_2DL_5B5665C69 | TraesCS2D02G404200 |
| *TaCDPK18-4AL* | Traes_4AL_FB1F95CD6 | TraesCS4A02G283400 |
| *TaCDPK18-4BL* | Traes_4BS_E01B5DAC9 | TraesCS4B02G030100 |
| *TaCDPK18-4DL* | Traes_4DS_63F7CF3CE | TraesCS4D02G027600 |
| *TaCDPK19-5AL* | Traes_5AL_2C0D0BFE0 | TraesCS5A02G426800 |

| | | |
|---|---|---|
| *TaCDPK19-5BL* | Traes_5BL_8B7A2A3FE | TraesCS5B02G428700 |
| *TaCDPK19-5DL* | Traes_5DL_E4E401B4C | TraesCS5D02G434800 |
| *TaCDPK20-5BS* | Traes_5BS_9FE1E88B8 | TraesCS5B02G109300 |
| *TaCDPK20-5DS* | Traes_5DS_8471D9E4E | TraesCS5D02G124000 |
| *TaCDPK21-6AL* | Traes_6AL_E6FD1E7AD | TraesCS6A02G261200 |
| *TaCDPK21-6BL* | Traes_6BL_AAB167E90 | TraesCS6B02G288600 |
| *TaCDPK21-6DL* | Traes_6DL_8828FB150 | TraesCS6D02G242000 |
| *TaCDPK22-5AS* | Traes_5AS_25D218AB9 | TraesCS5A02G107900 |
| *TaCDPK22-5BS* | Traes_5BS_4CBFAE917 | TraesCS5B02G115400 |
| *TaCDPK22-5DS* | Traes_5DS_69B96465C | TraesCS5D02G122800 |
| *TaCDPK23-5AL* | Traes_5AL_80176B533 | TraesCS5A02G138300 |
| *TaCDPK23-5BL* | Traes_5BL_CEF07DD751 | TraesCS5B02G137600 |
| *TaCDPK23-5DL* | Traes_5DL_701598D5F | TraesCS5D02G153800 |
| *TaCDPK24-1AL* | Traes_1AL_8D078BE99 | TraesCS1A02G309400 |
| *TaCDPK24-1BL* | Traes_1BL_28216FBDC | TraesCS1B02G320400 |
| *TaCDPK24-1DL* | Traes_1DL_F3CA101B0 | TraesCS1D02G308800 |
| *TaCDPK25-6AS* | Traes_6AS_CE8BAAE7A | TraesCS6A02G089400 |
| *TaCDPK25-6BS* | Traes_6BS_814D20B55 | TraesCS6B02G111800 |
| *TaCDPK25-Un* | Traes_6BS_814D20B55 | TraesCSU02G016900 |
| *TaCDPK26-4DL* | Traes_4DL_052000A28 | TraesCS4D02G318400 |
| *TaCDPK27-5AL* | Traes_5AL_F82860F37 | TraesCS5A02G490200 |
| *TaCDPK28-3AL* | Traes_3AL_0360C0D50 | TraesCS3A02G351300 |
| *TaCDPK28-3BL* | Traes_3B_C7F6BF696 | TraesCS3B02G383800 |
| *TaCDPK29-5AL* | Traes_5AL_C5F21415C | TraesCS5A02G298500 |
| *TaCDPK29-5BL* | Traes_5BL_DD7987EDE | TraesCS5B02G297700 |
| *TaCDPK29-5DL* | Traes_5DL_2D48BD7B0 | TraesCS5D02G305200 |
| *TaCDPK30-7AS* | Traes_7AS_830505028 | TraesCS7A02G267000 |
| *TaCDPK30-7BS* | Traes_XX_EB10C2A3C | TraesCS7B02G165200 |
| *TaCDPK30-7DS* | Traes_XX_13941845 | TraesCS7D02G267700 |

Table S4. The primers used for qRT-PCR, gene clone, and vector construction in this study

| Gene Name | Primer Sequences | Production/bp |
|---|---|---|
| **qRT-PCR for analysis of tissue and development expression level** | | |
| *TaCDPK1*(B) | Forward:5' GGTGGTGTTATTGGGATGGGTA 3'<br>Reverse:5' GCCCGAGGACAGAGATGGAT 3' | 229 |
| *TaCDPK2*(A) | Forward:5' GTGGGGCGAAGAACAATGAG 3'<br>Reverse:5' TCCACACAGGCATTTTACAAGAT 3' | 169 |
| *TaCDPK3*(D) | Forward:5' GACCGAACAAGGGGTAGCAC 3'<br>Reverse:5' CCGATACATTAGGCCAGGGT 3' | 77 |
| *TaCDPK4*(A) | Forward:5' CAACAGGGAGGCGAACCAT 3'<br>Reverse:5' ATCCAGCGTCTAAGATTCGGTT 3' | 265 |
| *TaCDPK5(B)* | Forward:5' TAGATTTCTGGTGCTGGTGTTTTAC 3'<br>Reverse:5' ATACACGCAAATTGGGTAACAGA 3' | 141 |
| *TaCDPK7*(D) | Forward:5' TGACGCTGATGGAAATGGGT 3'<br>Reverse:5' CGGATGATGGCATTGATAACC 3' | 210 |
| *TaCDPK8*(B) | Forward:5' CAGCGACAATGCACATGAATAGA 3'<br>Reverse:5' GTTATGTATCCGCTGTGGTCCTTAT 3' | 91 |
| *TaCDPK12*(D) | Forward:5' GCCCCATCATCCTCCTCAC 3'<br>Reverse:5' GCACCGCGACCTTTACCA 3' | 164 |
| *TaCDPK13*(A) | Forward:5' GCAACAGAACAGCAAGCGAT 3'<br>Reverse:5' AGGGAACCAGCAGAACCAGA 3' | 189 |
| *TaCDPK15*(D) | Forward:5' CGTTGTCGGAAGCCCCTAC 3'<br>Reverse:5' GCTCGTTCTCTGCCCAAAAT 3' | 137 |
| *TaCDPK16*(D) | Forward:5' AGACGCACCCGATACGCC 3'<br>Reverse:5' CCTCTTCCGATAAGCACCCAG 3' | 119 |
| *TaCDPK19*(D) | Forward:5' AACACCGCCAAACTCAAGGA 3'<br>Reverse:5' AGACGACGCTGCTGTGCC 3' | 214 |
| *TaCDPK20*(D) | Forward:5' TTGAACAAAGTTGAGAGGGAAGAC 3'<br>Reverse:5' ACATCCTCAATCCCAAACTCGT 3' | 125 |
| *TaCDPK21*(A) | Forward:5' CGCAGAGCGTCTAACAGCAC 3'<br>Reverse:5' CTATTGTCTGTGTCCATTGCCTC 3' | 225 |
| *TaCDPK22*(B) | Forward:5' CAATAGATTTTCCAACCACGCAT 3'<br>Reverse:5' GAAGAGCGGGGAGAAATGTGT 3' | 136 |
| *TaCDPK25*(A) | Forward:5' CCAAGATGACCCGACCTGTT 3'<br>Reverse:5' TCCAATAGTTCACCACCTTCACA 3' | 163 |
| *TaCDPK26*(D) | Forward:5' ATTACTACAATCGGTTCCCCAG 3'<br>Reverse:5' CTTGCCTCAGAATACCAGCG 3' | 150 |
| *TaCDPK27*(A) | Forward:5' ATGCCGATGGGATTGAAGAT 3'<br>Reverse:5' CCAACCCTTGCCTCAGAATAC 3' | 114 |
| *TaNOX7(A)* | Forward:5' ATATTCATGCGAGCGCATT3'<br>Reverse:5' AGAAGACTGTCCTAAGCCGAG 3' | 275 |
| *TaACTIN* (AB181991.1) | Forward:5'GAAGTGCTTTTGAAGAGTCGGT3'<br>Reverse:5'TTATTTCATACAGCAGGCAAGC3' | 207 |
| *TaGAPDH* (A0A1D6ATH8) | Forward:5'TTAGACTTGCGAAGCCAGCA3'<br>Reverse:5'AAATGCCCTTGAGGTTTCCC3' | 81 |
| **PCR for gene clone** | | |
| *TaNOX7(A)* | Forward:5' CGGAATTCTACTTCATCCGTTGTTCCGCACAA 3'<br>Reverse:5'AAAACTGCAGCTTTATTTACCTCTCAGGGCAATT3' | 2063 |
| *TaCDPK2(A)* | Forward:5' GATAAATCCATCCTCCCCAACG 3'<br>Reverse:5' ATCAGAATCCCCAGAAACACACAT ' | 2034 |
| *TaCDPK4*(A) | Forward:5' CCCAGTTCTTCCGTTCTCAT 3'<br>Reverse:5' CATCTATCTCCCTTTCCCTCTTA 3' | 1966 |
| *TaCDPK13*(D) | Forward:5' GGAAGGCAGGCTACAACAAACAC 3'<br>Reverse:5' CGACTCCCAATCCCACGGTAT 3' | 1922 |
| *TaCDPK16*(D) | Forward:5' CTTCCAAACTCCGAATCCATC 3'<br>Reverse:5' ATTCATGGGTATGTATGTGGA 3' | 1909 |
| *TaCRK20* (D) | Forward:5' ACGAACCGAGACCACCAATAA 3' | 2200 |

| | | |
|---|---|---|
| | Reverse:5' ATCACACTTCCCTTTCCTTTACAA 3' | |
| *TaCRK21*(B) | Forward:5' CCGCAACAAGTCTCACCCTC 3'<br>Reverse:5' CCACCAATTAGAATGCCCACA 3' | 1895 |
| **Vector construction for P1301-2*35S-NOX-GFP** | | |
| *TaNOX7*(A) | Forward:5' GCGGATCCATGGAGATGCCTGATATTGAAGCT 3'<br>Reverse:5' CGGTACCCTAGAAGTTCTCCTTGTGAAACTC 3' | 2718 |
| **Vector construction for P1301-2*35S-CDPK-Cherry** | | |
| *TaCDPK2A* | Forward:5' GCTCTAGAATGGGCAACGCATGCGGCGG 3'<br>Reverse:5' CGGGATCCGATTGCACCAGGTGCGTCTCTC 3' | 1690 |
| *TaCDPK4A* | Forward:5' GCTCTAGAATGCAGCCGGACGCGAGCGG 3'<br>Reverse:5' CGGGTACCGCTGTTGCTGGGATTCAAGATGTC 3' | 1555 |
| *TaCDPK13A* | Forward:5' GCTCTAGAATGGGCAACTGCTGCGCGGG 3'<br>Reverse:5' CGGGTACCTAGCATGACGTCGCGCCGTT 3' | 1562 |
| **Vector construction for nLUC** | | |
| *TaNOX7A* | Forward: 5' CGGGATCCATGGAGATGCCTGATATTGAAGCTGGC 3'<br>Reverse: 5' ACGCGTCGACGAAGTTCTCCTTGTGAAACTCAAACTTGGT 3' | 2718 |
| **Vector construction for cLUC** | | |
| *TaCDPK2A* | Forward:5' CGGGATCCGATGGGCAACGCATGCG 3'<br>Reverse:5' ACGCGTCGACCTAGATTGCACCAGGTGCGTCTCT 3' | 1696 |
| *TaCDPK4A* | Forward:5' CGGGGTACCATGCAGCCGGACGCGA 3'<br>Reverse: 5' ACGCGTCGACTTAGCTGTTGCTGGGATTCAAGATGT 3' | 1561 |
| *TaCDPK13A* | Forward: 5' CCCGGGGCGGTACCCGGGATCCGATGGGCAACTGCTGC 3'<br>Reverse:5' GAACGAAAGCTCTGCAGGTCGACCTATAGCATGACGTCGCGCCG 3' | 1603 |
| *TaCDPK16D* | Forward: 5' CGGGGATCCATGCAGCCGGACGCGA 3'<br>Reverse:5' ACGCGGTACCTCACTCTAGAACTAG 3' | 1618 |
| *TaCRK20D* | Forward:5' CCCGGGGCGGTACCCGGGATCCG ATGGGCAACGTCTGC 3'<br>Reverse:5' GAACGAAAGCTCTGCAGGTCGACTTAGCTATTTGACTTTATGTTCAAG 3' | 1921 |
| *TaCRK21B* | Forward: 5' CCCGGGGCGGTACCCGGGATCCG ATGGGCAACACGTGCG 3'<br>Reverse:5' GAACGAAAGCTCTGCAGGTCGAC TCATTCAGTCATGCTTATATTCAGG 3' | 1699 |
| **Vector construction for pSPYCE** | | |
| *TaNOX7A* | Forward:5' AGAGAACACGGGGGACTCTAGAATGGAGATGCCTGATATTG 3'<br>Reverse:5' ATGGGTACATCCCGGGAGCGGTACCGTTTCACAAGGAGAACTTC 3' | 2747 |
| **Vector construction for pSPYNE** | | |
| *TaCDPK2A* | Forward:5' AGAGAACACGGGGGACTCTAGAATGGGCAACGCATGCGGC 3'<br>Reverse:5' TGCTCCATCCCGGGAGCGGTACCGATTGCACCAGGTGCGTC 3' | 1719 |
| *TaCDPK4A* | Forward:5' AGAGAACACGGGGGACTCTAGAATGCAGCCGGACGCGA 3'<br>Reverse:5' TGCTCCATCCCGGGAGCGGTACCGCTGTTGCTGGGATTC 3' | 1584 |
| *TaCDPK13A* | Forward:5' AGAGAACACGGGGGACTCTAGAATGGGCAACTGCTGCGC 3'<br>Reverse:5' TGCTCCATCCCGGGAGCGGTACCTAGCATGACGTCGCGCCGT 3' | 1599 |
| *TaCDPK16D* | Forward:5' AGAGAACACGGGGGACTCTAGAATGGGCCAATGCTGCGCC 3'<br>Reverse:5' TGCTCCATCCCGGGAGCGGTACCTCACTCTAGAACTAGATCA 3' | 1644 |
| **Vector construction for P1301-2*35S-NOX-GFP** | | |
| *TaNOX7*(1047)* | Forward:5' GCGGATCCATGGAGATGCCTGATATTGAAGCTGGC 3'<br>Reverse:5' CGGGGTACCCTTCCAGTTTTCCTCAACAAAGTACAAAAAC 3' | 1064 |
| **Vector construction for P1300-2*35S-CDPK-6*MYC** | | |
| *TaCDPK2A* | Forward:5' GGGTTCGAAATCGATGGATCCTAATGGGCAACGCATGCGGC 3'<br>Reverse:5' GGGAAATTCGAGCTCACTAGTCTAGATTGCACCAGGTGCGT 3' | 1721 |
| *TaCDPK4A* | Forward:5' GGGTTCGAAATCGATGGATCCTAATGCAGCCGGACGCGAGC 3'<br>Reverse:5' GGGAAATTCGAGCTCACTAGTTTAGCTGTTGCTGGGATTC 3' | 1586 |
| *TaCDPK13A* | Forward:5' GGGTTCGAAATCGATGGATCCTAATGGGCAACTGCTGCGC 3'<br>Reverse:5' GGGAAATTCGAGCTCACTAGTCTATAGCATGACGTCGCGC 3' | 1598 |

* *TaNOX7*$_{(1047)}$ represents the gene sequence from the start codon ATG to the 1047th base of *TaNOX7*.

Table S5. Homology of CDPK family proteins among different species

| Genes in wheat | | Genes in Other species | | Identification |
|---|---|---|---|---|
| Genes names | Gene ID | Genes names | Gene ID | (%) |
| TaCDPK1 | TraesCS4B02G321800.1 | OsCDPK7 | Os03t0128700-01 | 82.7% |
| | | AtCPK6 | AT2G17290.2 | 70.3% |
| TaCDPK2 | TraesCS2A02G456100.1 | OsCDPK13 | Os04t0584600-01 | 95.9% |
| | | AtCPK5 | AT4G35310.1 | 87.3% |
| | | CsCDPK26 | KU201349 | 86.7% |
| TaCDPK4A | TraesCS4A02G206200.2 | OsCDPK24 | Os11t0171500-01 | 95.4% |
| | | ZmCPK11 | Zm00001eb091710 | 92.5% |
| | | AtCDPK2 | AT1G35670.1 | 77.5% |
| TaCDPK4B | TraesCS4B02G109800.2 | ZmCPK11 | Zm00001eb091710 | 92.5% |
| TaCDPK5A | TraesCS2A02G223100.2 | AtCPK23 | AT4G04740.2 | 70.4% |
| TaCDPK6A | TraesCS2A02G239900.1 | AtCPK16 | AT2G17890.1 | 81.2% |
| | | AtCPK28 | AT5G66210.4 | 80.7% |
| TaCDPK7A | TraesCS2A02G199700.1 | StCDPK32 | XP_006351224.1 | 79.3% |
| | | AtCDPK1 | AT1G18890.1 | 70.8% |
| TaCDPK8A | TraesCS3A02G224900.1 | OsCDPK1 | Os01t0622600 | 94.2% |
| | | AtCPK3 | AT4G23650.1 | 79.8% |
| TaCDPK9A | TraesCS2A02G248200.3 | OsCDPK17 | Os07t0161600-01 | 91.6% |
| TaCDPK17A | TraesCS2A02G407200.1 | OsCDPK12 | Os04t0560500-00 | 89.6% |
| | | AtCPK21 | AT4G04720.2 | 67.3% |
| TaCDPK20B | TraesCS5B02G109300.1 | CsCDPK20 | KU201348 | 79.8% |
| | | GmCDPK3 | GLYMA_02G192700 | 78.5% |
| TaCDPK20D | TraesCS5D02G124000.1 | AtCPK1 | AT5G04870.1 | 81.8% |
| | | CsCDPK20 | KU201348 | 79.4% |
| | | GmCDPK3 | GLYMA_02G192700 | 77.9% |
| | | AtCDPK4 | AT4G09570.1 | 66.9% |
| TaCDPK21A | TraesCS6A02G261200.1 | AtCDPK2 | AT1G35670.1 | 68.6% |
| TaCDPK23D | TraesCS5D02G153800.1 | AtCDPK32 | AT3G57530.1 | 54.8% |
| TaCDPK26D | TraesCS4D02G318400.1 | AtCPK5 | AT4G35310.1 | 67.9% |
| | | AtCDPK9 | AT5G23580.1 | 60.2% |
| TaCDPK27A | TraesCS5A02G490200.1 | ZmCPK1 | GRMZM2G032852 | 83.0% |
| | | OsCDPK21 | Os08t0540400-01 | 89.0% |
| TaCDPK30A | TraesCS7A02G267000.1 | ZmCPK32 | GRMZM2G33266 | 85.9% |
